# Bacteriophage GIL01 gp7 interacts with host LexA repressor to enhance DNA binding and inhibit RecA-mediated auto-cleavage

**DOI:** 10.1093/nar/gkv634

**Published:** 2015-07-02

**Authors:** Nadine Fornelos, Matej Butala, Vesna Hodnik, Gregor Anderluh, Jaana K. Bamford, Margarita Salas

**Affiliations:** 1 Department of Biological and Environmental Science and Nanoscience Center, University of Jyvaskyla, Centre of Excellence in Biological Interactions, PO Box 35, F-40014 Jyvaskyla, Finland; 2 Instituto de Biología Molecular ‘Eladio Viñuela’ (CSIC), Centro de Biología Molecular ‘Severo Ochoa’ (CSIC-Universidad Autónoma de Madrid), Cantoblanco, 28049 Madrid, Spain; 3 Department of Biology, Biotechnical Faculty, University of Ljubljana, Večna pot 111, 1000 Ljubljana, Slovenia; 4 National Institute of Chemistry, Hajdrihova 19, 1000 Ljubljana, Slovenia

## Abstract

The SOS response in Eubacteria is a global response to DNA damage and its activation is increasingly associated with the movement of mobile genetic elements. The temperate phage GIL01 is induced into lytic growth using the host's SOS response to genomic stress. LexA, the SOS transcription factor, represses bacteriophage transcription by binding to a set of SOS boxes in the lysogenic promoter *P1*. However, LexA is unable to efficiently repress GIL01 transcription unless the small phage-encoded protein gp7 is also present. We found that gp7 forms a stable complex with LexA that enhances LexA binding to phage and cellular SOS sites and interferes with RecA-mediated auto-cleavage of LexA, the key step in the initiation of the SOS response. Gp7 did not bind DNA, alone or when complexed with LexA. Our findings suggest that gp7 induces a LexA conformation that favors DNA binding but disfavors LexA auto-cleavage, thereby altering the dynamics of the cellular SOS response. This is the first account of an accessory factor interacting with LexA to regulate transcription.

## INTRODUCTION

The SOS response is a highly conserved mechanism by which bacteria identify and repair DNA damage inside the cell (1). The two key components of the SOS response are the recombinase RecA and the global transcription factor LexA (2,3). Under normal growth conditions, LexA represses transcription of numerous DNA damage-inducible genes by binding to an upstream DNA sequence termed the SOS box. Upon DNA damage, RecA bound to single-stranded DNA stimulates the autocatalytic cleavage of LexA, thereby lifting repression of LexA-regulated genes, including *recA* and *lexA* itself. LexA eventually restores the repressed state as the DNA is repaired and levels of activated RecA drop.

In *Escherichia coli*, LexA directly regulates some 30 promoters that direct the expression of genes involved in repairing or replicating damaged DNA (4,5). LexA is increasingly associated with regulating functions that are unrelated to cell rescue (6). Examples are the movement of mobile elements (7,8), the propagation of virulence factors (9,10) and the lytic cycle of temperate phages (11–14). Although LexA is the master repressor of the SOS response, an in vivo study of LexA binding sites in *E. coli* identified about 20 novel targets that lacked a recognizable LexA binding motif and failed to be bound by LexA in vitro. These sites are likely to require an accessory factor for LexA binding (15).

Bacteriophage GIL01 infects the insect pathogen *Bacillus thuringiensis* and establishes a stable, quiescent residence within its host (16). While this lysogenic state is particularly stable, GIL01 can efficiently switch to the lytic cycle in response to DNA-damaging treatments. This is the case for many temperate phages, best exemplified by phage lambda, which take advantage of a conserved cellular DNA-damage signaling pathway in order to escape a potentially doomed host. Unlike most temperate phages, however, GIL01 does not code for its own stress-sensitive repressor. Instead, our results showed that stable lysogeny relies on the host transcription factor LexA (17).

**Figure 1.**
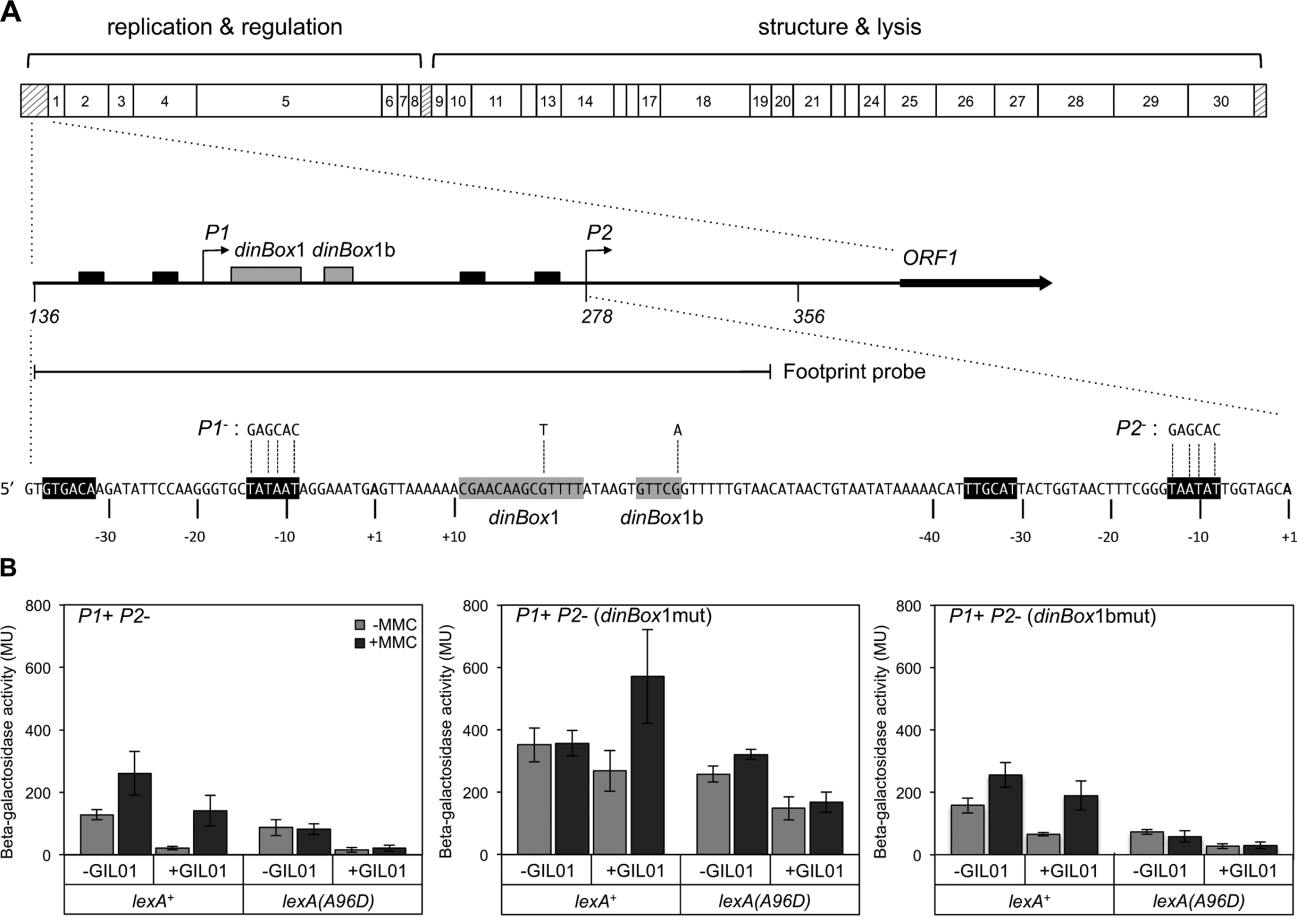
*P1* is regulated by host LexA. (**A**) Genetic map of GIL01 with the *P1–P2* promoter region enlarged below. Transcription start sites of the tandem promoters *P1* and *P2* are depicted with angled arrows (middle schematic) and shown in boldface letters (+1; bottom schematic). The −35 and −10 promoter consensus sequences are represented in black and LexA binding sites *dinBox*1 and *dinBox*1b are shown in gray. The beginning of ORF1 is indicated by a full arrow. Nucleotide substitutions used to generate promoter and SOS box mutant fusions to the *lacZ* gene are indicated by stippled lines above the respective promoter sequence. The boundaries of probes used in DNase I footprint experiments (Figures 2C and 4) are delineated below the map. (**B**) A *P1*^+^*P2*^−^-*lacZ* fusion was generated by inactivating promoter *P2* with four substitutions to its −10 sequence (left panel). The resulting *lacZ* fusion was then used to generate single nucleotide mutations in *dinBox*1 (middle panel) or *dinBox*1b (right panel) that are expected to eliminate or greatly reduce LexA binding to the respective site. Beta-galactosidase activity was measured in both *lexA*^+^ and *lexA(A96D)* phage-cured strains (−GIL01) and lysogen strains (+GIL01), under normal growth conditions (−MMC) and under SOS-inducing conditions (+MMC). Activities (Miller units) are the average of at least three independent experiments. MMC50 (0.05 µg/ml) is a mitomycin C concentration sufficiently high to elicit an SOS response (Supplementary Figure S1).

The GIL01 15-kb linear dsDNA genome does not integrate into the host chromosome during lysogeny. All predicted ORFs in GIL01 are transcribed in the same left-to-right direction, possibly as a means to minimize interference between gene transcription and genome replication. The GIL01 genome is divided into two main modules: the left 5-kb of the genome are dedicated to genome replication and regulation while the right 10-kb code for virus structural and lytic functions (Figure 1A). Accordingly, two promoter regions were identified upstream of each module. To the left, *P1* and *P2* direct the expression of immunity and replication genes whereas the internal *P3* promoter controls lytic gene expression (17). The tandem organization of *P1* and *P2* suggests that both promoters likely display distinct strengths and are differentially regulated.

Using a genetic approach, we identified *dinBox*1, a 14-bp long sequence in the *P1* promoter region that is similar to the consensus LexA binding site in *Bacillus subtilis* (CGAAC(n)_4_GTTCG) (18,19). Mutants in *dinBox*1 were unable to establish a lysogenic state, suggesting that *dinBox*1 functions as a LexA binding site and that LexA binding is important for GIL01 lysogeny (17). This was verified by generating a LexA-noncleavable mutant host, *lexA(A96D)*, that is deficient for the auto-proteolysis reaction and thereby unable to induce the SOS response. GIL01 could not be induced in the *lexA(A96D)* host, confirming that the host SOS pathway regulates GIL01 development. This study also identified two small GIL01 gene products, gp1 and gp7, required for stable lysogen formation. Gp1 is very likely a DNA binding protein but a possible function for gp7 was not found.

In this work, we report that host LexA directly regulates phage gene expression from promoter *P1*. In addition to binding to the predicted SOS site *dinBox*1, purified *B. thuringiensis* LexA also binds to an adjacent non-canonical sequence termed *dinBox*1b that appears to contain only one LexA half-site (Figure 1A). Point mutations in the conserved bases in either *dinBox*1 or *dinBox*1b significantly reduce LexA affinity and binding is nearly eliminated to a doubly mutated probe. While the *dinBox*1 and *dinBox*1b sites possibly overlap, our experiments indicate that LexA binds to both sites in an independent, non-cooperative manner. In vitro, LexA binding to *dinBox*1b is unstable unless the small phage-coded protein, gp7, is also provided. Gp7 alone does not bind to promoter DNA but specifically interacts with immobilized LexA in the absence of DNA. Interestingly, the gp7-enhancing effect was also observed with other cellular LexA targets, such as the SOS box directly upstream of the *lexA* gene. We provide evidence that gp7 stabilizes LexA binding to bacteriophage as well as host SOS boxes. Most importantly, we show that gp7 also interferes with the RecA-mediated LexA auto-cleavage reaction. Our results identify for the first time, a factor that directly interacts with the major SOS transcription factor LexA to modulate the SOS response.

## MATERIALS AND METHODS

### Bacterial strains and culture conditions

*Escherichia coli* strain DH5α was routinely used for plasmid constructions destined to be transformed into *B. thuringiensis*. Strain XL10-Gold (Stratagene) was used for site-directed mutagenesis, and strains M15 [pREP4] (Qiagen) and BL21 (DE3) pLysE were used for protein expression, as described.

*Bacillus thuringiensis* serovar *israelensis* isogenic strains GBJ002 and GBJ338 were used as GIL01-cured and GIL01-lysogenic hosts, respectively (17). *B. thuringiensis* strains GBJ396 [*lexA(A96D)*] and GBJ499 [*recA*::pMutin4] are GBJ002 derivatives mutated in the *lexA* and *recA* genes, respectively (17).

*Escherichia coli* and *B. thuringiensis* strains were grown in L-broth at 37°C and 30°C, respectively. To induce DNA damage, mitomycin C (0.05 μg/ml) was added to one-half of mid-log phase cultures for 1 h at 30°C. To induce gp7 expression in *Bacillus*, 0.1 mM isopropyl β-d-thiogalactopyranoside (IPTG) was added to one-half of the cultures and the other half was incubated in parallel to provide an uninduced control. After 1 h of induction, cultures were stressed by addition of mitomycin C (0.05 μg/ml) for an additional hour.

### Plasmid construction

The primers used in this study are listed in Supplementary Table S1.

A *P1–P2* promoter fusion to *lacZ* in the low-copy-number shuttle vector pHT304–18Z was described previously (17). To examine expression from *P1* alone, transcription from *P2* was eliminated using QuikChange Lightning site-directed mutagenesis (Stratagene) to mutate the -10 site from 5′-TAATAT to 5′-GAGCAC, generating pNF543. In order to generate a promoter-free control, pNF543 was used as template to eliminate *P1* by replacing its −10 site from 5′-TATAAT to 5′-GAGCAC. Mutations within the LexA binding sites *dinBox*1 and *dinBox*1b were similarly generated using pNF543 as a template. The promoter regions upstream of *lexA* (coordinates 3 623 933 through 3 624 142 in *B. cereus* strain G9842; GenBank accession number NC_011772) and *recA* (coordinates 3 720 030 to 3 720 243 in strain G9842) were PCR amplified and the respective products were purified, restricted and ligated into the HindIII–BamHI-digested pHT304–18Z vector. The resulting *lacZ* promoter constructs were transformed into strains GBJ002 [l*exA*^+^] and GBJ396 [*lexA(A96D)*] for analysis of β-galactosidase expression. Transformation was done by electroporating *Bacillus* electrocompetent cells with 0.5 μg plasmid DNA in 2 mm cuvettes at 25 μFa, 1.4 kV and 400 Ω.

The ORF7 expression plasmid pDG7 was as previously described (17). pDG7 was transformed into GBJ002 [l*exA*^+^] strains harboring *lacZ* promoter fusions. Control strains contained *lacZ* promoter fusions along with the empty vector pDG148.

*Bacillus lexA* (coordinates 3 624 082 through 3 624 714 in strain G9842), ORF1 (GIL01 coordinates 356 through 532; GenBank accession number AJ536073) and ORF7 (GIL01 coordinates 4564 through 4716) were PCR amplified using primers flanked by BamHI and HindIII restriction sites, digested and cloned into expression vector pQE-30 (Qiagen), downstream of the IPTG-inducible T5 promoter.

*Bacillus recA* (coordinates 3 719 029 through 3 720 060 in strain G9842) was PCR amplified using primers flanked by BamHI and MluI restriction sites, digested and cloned into expression vector pET8c (Novagen) to generate pRecABc. The constructs were verified by DNA sequencing. All four genes were expressed as recombinant proteins with an N-terminal His_6_ epitope tag.

### Overexpression and purification of His_6_-LexA and His_6_-gp7

*Escherichia coli* M15 [pREP4], containing the *lexA*, gp1 or gp7 gene fusion, was grown in 500 ml L-broth containing ampicillin (150 μg/ml) and kanamycin (25 μg/ml). Protein expression was induced at an OD_600_ of 0.7 by the addition of 1 mM IPTG. After 3 h at 30°C, the cells were harvested by centrifugation, resuspended in 50 mM NaH_2_PO_4_ and 0.3 M NaCl (buffer A) containing 10 mM imidazole, and lysed by addition of lysozyme to 0.1 mg/ml. His_6_-LexA, His_6_-gp1 and His_6_-gp7 were purified from cell lysates by passing them over a Ni-nitrilotriacetic acid (NTA) column pre-equilibrated with buffer A containing 10 mM imidazole. The column was washed extensively with the same buffer, followed by buffer A containing 20 mM imidazole. His-tagged proteins were eluted with buffer A containing 250 mM imidazole. Fractions containing the over-expressed proteins, as determined by SDS-PAGE, were concentrated on Amicon Ultra-15 centrifugal filter units with a 3-kDa (His_6_-gp1 and His_6_-gp7) or 10-kDa (His_6_-LexA) cut-off (Millipore), buffer exchanged into 50 mM Tris–HCl, pH 7.5, 1 mM EDTA, 50 mM NaCl, 7 mM β-mercaptoethanol and stored in 50% glycerol at –80°C.

### Preparation of recombinant RecA protein

To induce the synthesis of RecA protein, an overnight culture of *E. coli* BL21 (DE3) pLysE strain harboring pRecABc was grown in 500 ml L-broth containing ampicillin (100 μg/ml) and chloramphenicol (25 μg/ml), to an OD_600_ of 0.6 when 0.8 mM IPTG was added to the culture. After 4 h, the cells were harvested and the cell pellet was stored at –20°C. The recombinant RecA protein was synthesized insoluble in inclusion bodies. The cells were resuspended at 1 g wet mass/ml in lysis buffer (50 mM NaH_2_PO_4_, 0.3 M NaCl, 10 mM imidazole, pH 8.0), supplemented with lysozyme (0.5 mg/ml), DNAse (10 μg/ml), RNAse (20 μg/ml), benzamidine (1 mM), AEBSF (0.5 mM), PMSF (0.5 mM) and sonicated (vibracell, Sonics) at 40% amplitude 6 times for 20 s. The homogenate was centrifuged at 26 000 × *g* for 30 min at 4°C. Inclusion bodies were washed in 20 mM Tris–HCl, 0.5 M NaCl, 2% (v/v) Triton X-100, pH 8.0, sonicated and centrifuged twice and stored at -20°C. The RecA protein was dissolved in 20 mM Tris–HCl, 0.5 M NaCl, 6 M guanidinium chloride, 5 mM imidazole, 5 mM β-mercaptoethanol, pH 8.0 (2 ml buffer/g inclusion body wet mass) by stirring for 1 h at 4°C. To refold the recombinant RecA, the dissolved protein was diluted 100-fold in 20 mM Tris–HCl, 0.5 M NaCl, 5 mM imidazole, 5 mM β-mercaptoethanol, pH 8.0, at 4°C. The refolded protein was concentrated using Amicon Ultra-15 centrifugal filter units with a 10-kDa cut-off (Millipore). The concentration of recombinant RecA protein was determined using NanoDrop 1000 (Thermo Scientific) and the extinction coefficient at 280 nm was 15 930/M/cm.

### RecA*-dependent LexA repressor self-cleavage assay

Recombinant RecA (10 μM) was activated with the S1 primer (2.2 μM) (Supplementary Table S1), 2 mM MgCl_2_ and 1 mM ATP-γ-S as described (20) for 2 h on ice. The LexA repressor (2.1 μM) was either free or pre-incubated with gp7 (8.6 μM) for 3 min at 37°C. Cleavage reactions were performed in 20 mM Tris (pH 7.4), 5 mM MgCl_2_, 1 mM ATP-γ-S, 1 mM DTT, 60 mM NaCl, 0.6 mM imidazole, 1 mM β-mercaptoethanol, 0.15 mM EDTA and 15% glycerol at 37°C. The last five components came from the addition of RecA and gp7 storage buffers and were also added to reaction mixtures without gp7. The reaction time course was initiated by the addition of activated RecA filaments (RecA at 2 μM in 20 μl reaction mixtures) or by the addition of inactive RecA filaments formed in the absence of ssDNA and ATP-γ-S. All reactions were stopped by addition of NuPAGE LDS sample buffer (Invitrogen). Samples were analyzed on 15% SDS-PAGE gels and stained by Page blue protein stain (Thermo Scientific). The resolved bands were quantified as described (21).

### Surface plasmon resonance assays

SPR measurements were performed on a Biacore T100 (GE Healthcare) at 25°C. The streptavidin sensor chip SA (GE Healthcare) was washed with a 60 s injection of 50 mM NaOH and 1 M NaCl and equilibrated in SPR buffer (25 mM Tris–HCl [pH 7.3], 140 mM NaCl, 5 mM EDTA, 2 mM dithiothreitol (DTT), 0.1 mg/ml BSA, 0.005% surfactant P20). Approximately 30 response units (RU) of 3′-biotinylated S1 primer (Supplementary Table S1) was immobilized on the flow cells of the streptavidin chip. To prepare double-stranded DNA fragments with *P1* promoter or *lexA* operator sequences, or their derivatives with modified SOS boxes, complementary primers denoted as primer name_u and primer name_d in 20 mM Tris–HCl (pH 7.5), 140 mM NaCl were mixed in a 1:1.5 (mol:mol) ratio, respectively. Primers were annealed using a temperature gradient from 94°C to 4°C (∼1.5 h) in a PCR machine (Eppendorf). The resulting 40–42-bp duplex DNA, with a 15 nucleotide single-strand 5′ overhang complementary to the SPR chip-immobilized S1 primer, was passed for 1–5 min at 2 μl/min across the flow cell 2, immobilizing 30 to 60 RU of DNA fragment. The interaction between His-tagged LexA, gp7 or LexA pre-incubated with gp7 for a few minutes at room temperature, and the chip-immobilized DNAs was analyzed by injecting solutions of the desired protein concentrations in SPR buffer at 100 μl/min. Regeneration of the sensor surface was performed with 50 mM NaOH for 10 s.

To study the kinetics of the LexA-gp7 interaction, His-tagged gp7 or LexA in 7 mM Na_2_HPO_4_ (pH 4), 12.5 mM KH_2_PO_4_, 140 mM NaCl were covalently coupled to the surface of the sensor flow cell activated with 0.4 M 1-ethyl-3-(3-dimethylaminopropyl)carbodiimide and 0.1 M *N*-hydroxysuccinimide. Excess reactive groups were blocked with ethanolamine. Either His-tagged LexA, gp1 or gp7 proteins were used as analytes. Kinetic measurements were performed using sensor chip CM5 (GE Healthcare) at 25°C at a flow rate of 30 μl/min in SPR buffer. Regeneration of the sensor surface was performed with 1 mM NaOH for 15 s.

### Electrophoretic mobility shift assay

**Figure 2.**
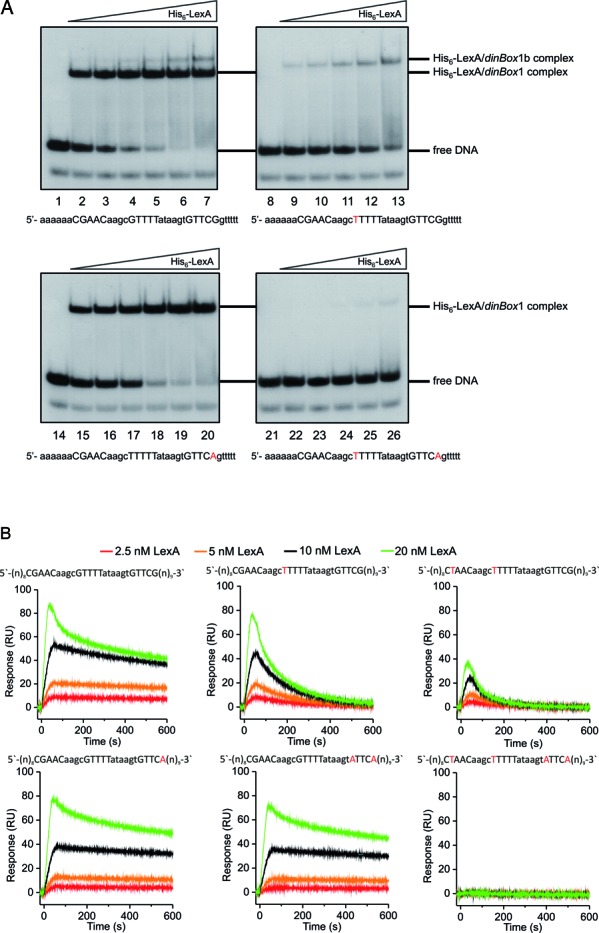

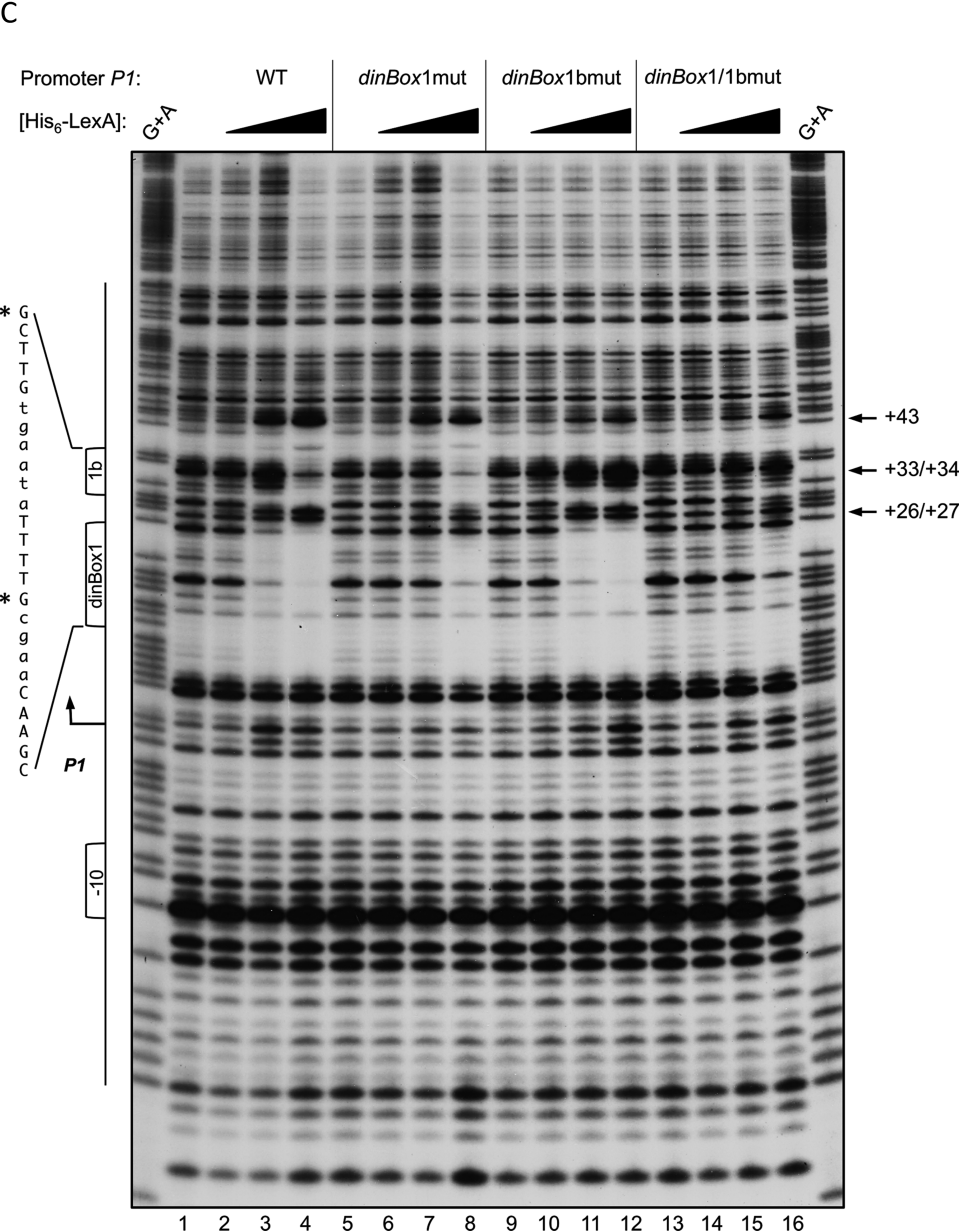
LexA binds to *dinBox*1 and to half-site *dinBox*1b. (**A**) Band shift assays of His_6_-LexA binding to probes encompassing *dinBox*1 and *dinBox*1b. Nucleotide sequences are detailed under each panel and positions that are known to be important for LexA binding are shown in uppercase letters. Mutations are shown in red. The upper strand of each probe was 5′ end-labeled prior to hybridization with the unlabeled complementary bottom strand. In each panel, the His_6_-LexA concentrations are 0, 0.25, 0.5, 1, 2, 8 nM and 16 nM (lanes 7 and 20). Labeled but non-hybridized oligonucleotides are visible at the bottom of each gel. (**B**) SPR analysis of the His_6_–LexA interaction with immobilized DNA fragments containing wild-type or mutated *dinBox* sites. His_6_–LexA in a concentration range of 2.5–20 nM was injected across the chip-immobilized DNA fragments (∼50 RU) for 60 s at a flow rate of 100 μl/min. The dissociation phase was followed for 540 s. Protein concentrations are denoted above the sensorgrams and operator sequences are presented above each graph with mutated nucleotides shown in red. The experiments were performed in triplicate and representative sensorgrams are shown. RU, response units. (**C**) DNase I footprint analysis of LexA binding to *dinBox*1 and *dinBox*1b. DNA fragments extending from positions −39 to +176 relative to the start of *P1* transcription (see Figure 1A) were ^32^P-labeled at the 5′-end of the top strand, incubated with His_6_-LexA, and digested with DNase I. Cleavage products were separated on an 8% sequencing gel. G+A marker lanes contain the same wild-type probe chemically cleaved at guanosine and adenosine residues. The four probes used for footprint analysis contained: the wild-type *P1* promoter sequence, a mutation in *dinBox*1, a mutation in *dinBox*1b or mutations in both *dinBox*1 and *dinBox*1b (see Figure 1A for SOS box nucleotide sequences). The first lane corresponding to each probe is labeled DNA without added protein. The following His_6_-LexA protein concentrations were used: 20, 80 and 320 nM. SOS box sequences are indicated in the left margin and the nucleotide positions that were substituted to generate the *dinBox*1 and *dinBox*1b mutated probes are indicated by an asterisk. The *P1* transcription start site is denoted with an angled arrow and the −10 sequence is indicated. Arrows in the right margin indicate the positions of DNase I hypersensitivity sites.

**Figure 3.**
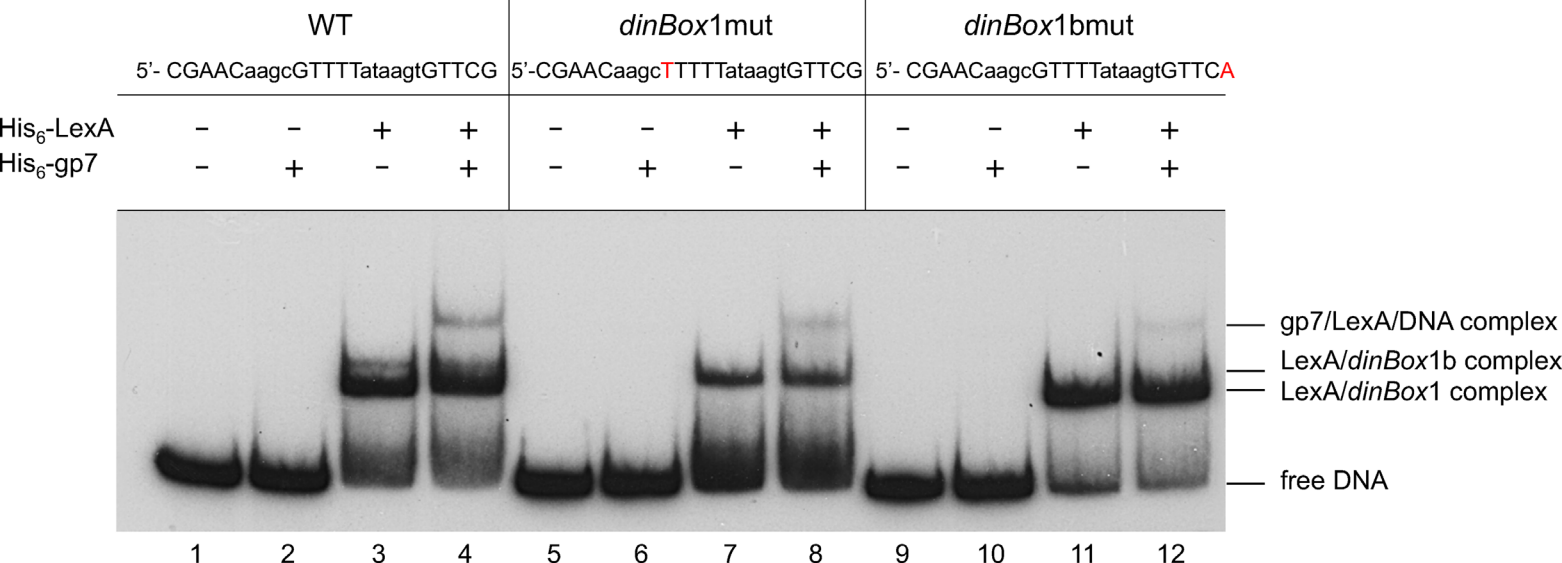
Gp7 forms a complex with LexA bound to the *dinBox* sites. Electrophoretic mobility shift assay of His_6_–LexA (8 nM) and His_6_–gp7 (340 nM) binding to 5′ end-labeled DNA probes extending from −25 to +176 relative to the start of *P1* transcription. The three probes used are identical except for single nucleotide mutations (shown in red) in *dinBox*1 (lanes 5–8) and *dinBox*1b (lanes 9–12) that eliminate LexA binding to the respective site.

Probes described in Figure 3 were amplified by PCR from GBJ338 genomic DNA, gel-purified and 5′-end labeled with [γ-^32^P] ATP and T4 polynucleotide kinase (NEB). Probes described in Figure 2A were constructed by hybridizing oligonucleotides, in which one nucleotide was 5′-end labeled and gel-purified prior to hybridization. Binding assays were performed by incubating different amounts of His_6_-LexA, in the presence or absence of 340 nM His_6_-gp7, with approximately 1 nM radiolabeled duplex oligonucleotides or 0.25 nM radiolabeled 200-bp PCR products in binding buffer (80 mM NaCl, 25 mM Tris [pH 7.5], 5 mM EDTA, 2 mM DTT, 0.1 mg/ml BSA, 0.05 μg/μl poly(dI-dC)). Reactions were incubated for 30 min at room temperature prior to loading directly onto native 6% (w/v) polyacrylamide gels. Electrophoresis was carried out at room temperature for 1.5 h at 30 mA using 1× TAE as the running buffer. Gels were dried directly to blotting paper and exposed to autoradiographic film.

### DNase I footprint analysis

Probes for DNase I footprints were generated by PCR using primers flanking the *P1* promoter region (Figure 1A). The primer complementary to the template strand was 5′-end labeled with [γ-^32^P] ATP and T4 polynucleotide kinase (NEB). PCR amplification products were purified from 1.2% agarose gels using the QIAquick Gel Extraction Kit (Qiagen). Binding reactions with varying amounts of purified His_6_–LexA or His_6_–LexA plus His_6_–gp7 were performed using the same binding conditions as for the gel-shift assays in a total volume of 20 μl. Reactions were incubated for 30 min at room temperature followed by addition of DNase I (0.1 U) (Promega) for 4 min. The DNase I reaction was terminated by the addition of 20 mM EDTA, 50 mM NaCl, 0.1 mg/ml tRNA and 1% SDS. The DNA products were ethanol precipitated, resuspended in loading buffer (50% formamide, 50% ddH_2_O, 0.01% xylene cyanol, 0.01% bromophenol blue) and heated at 90°C for 1–2 min prior to loading onto 8% sequencing gels (9 M urea). An A+G ladder was made by formic acid modification of the radiolabeled DNA, followed by piperidine cleavage (22).

### β-Galactosidase assays

β-Galactosidase assays were performed as described previously (17). *Bacillus* strains transformed with *lacZ* fusion plasmids were grown to exponential phase in L-broth containing 10 μg/ml erythromycin, at which point MMC (0.05 μg/ml) was added to one-half of the cultures. Cultures were incubated for one additional hour, and samples (20 μl) were taken and assayed as described. In strains where pDG7 was also present, 10 μg/ml kanamycin was included in the cultures. To induce gp7 expression from the inducible *spac* promoter, 0.1 mM IPTG was added to the cultures 1 h prior to MMC induction.

### RESULTS

#### Transcription from promoter *P1* is regulated by host LexA and phage-borne factors

We previously showed that LexA controlled GIL01 gene transcription by fusing promoters *P1* and *P2* to the *E. coli lacZ* reporter gene and analyzing LacZ expression in *B. thuringiensis* (17). A *P1–P2–lacZ* fusion was tightly repressed in a lysogen and expression could be induced ∼4-fold in SOS conditions. Accordingly, there was no induction of transcription from the same promoter fusion in an SOS-deficient *lexA(A96D)* host, indicating that LexA regulates *P1–P2* expression, most likely by directly binding to the predicted SOS box *dinBox*1 (Figure 1A). Considering the close proximity of *dinBox*1 to *P1*, centered at +17 from the start of *P1* transcription, we investigated the possibility that LexA regulates expression from *P1*. For that, we inactivated *P2* by replacing its -10 TAATAT box with the sequence GAGCAC to generate a *P1^+^P2^−^* promoter fusion. We also constructed a control fusion, *P1*^−^ *P2*^−^, in which both the *P1* and *P2* promoters were inactivated in a similar manner. As expected, this fusion did not show promoter activity in *B. thuringiensis* (5.65 ± 4.13 Miller units), indicating that the −10 box substitutions abolish promoter activity from both *P1* and *P2*. Figure 1B (left panel) shows that in a host lacking GIL01, *P1^+^P2^−^* activity is induced ∼2-fold in stress conditions (+MMC). The same *P1*^+^*P2*^−^-*lacZ* fusion is strongly repressed in a GIL01 lysogen (5.7-fold), and expression is lifted to normal levels upon SOS induction (+MMC). As expected, *P1^+^P2^−^* activity is repressed in a non-inducible *lexA(A96D)* host and cannot be induced in stress conditions, regardless of the presence or absence of a resident phage. Moreover, *P1^+^P2^−^* repression is noticeably stronger in the *lexA(A96D)* GIL01-lysogen than in the phage-cured host. These results identified *P1* as the LexA-repressed promoter.

To confirm that *dinBox*1 is the target of LexA-mediated regulation, we introduced a single nucleotide substitution at a conserved position that is known to be important for LexA binding (18). As can be seen from Figure 1B (middle panel), the *dinBox*1-mutated *P1* fusion shows high constitutive expression in a non-lysogen and is no longer SOS-inducible by MMC treatment, suggesting that LexA no longer recognizes this mutated *dinBox*. However, the same fusion is moderately repressed and induced in a GIL01 lysogen strain, indicating that *P1* is also regulated by unknown phage factors in an SOS-dependent fashion. Introduction of this fusion into the LexA(A96D) strain blocks this induction, indicating that this phage-dependent repression must somehow require LexA binding to *dinBox*1. These observations lead us to investigate a hypothesis that unknown phage-encoded factors are somehow ‘assisting’ host LexA in repressing *P1* expression in lysogens.

#### LexA binds to *dinBox*1 and to a downstream non-canonical site termed *dinBox*1b

To investigate the binding of LexA to *dinBox*1, *B. thuringiensis* LexA was over-expressed in *E. coli* as a 6xHistidine N-terminal fusion protein and used in electrophoretic mobility shift assays (EMSA) with radiolabeled oligonucleotide probes containing the *P1* promoter region. As shown in Figure 2A, His_6_–LexA formed a doublet with the wild-type probe (Figure 2A, lanes 1–7), consistent with the existence of two LexA binding sites in our DNA probe. Inspection of the probe sequence revealed a second potential binding site containing only one conserved LexA-binding ‘half-site’, termed *dinBox*1b. A mutant probe carrying a single G-T mutation at a highly conserved position in *dinBox*1 eliminated the faster-migrating form but retained the slower-migrating shifted form in EMSA experiments (Figure 2A, lanes 8–13). The slower-migrating complex was evident only at high His_6_-LexA concentrations and appeared to breakdown during gel electrophoresis, suggesting that LexA binds to *dinBox*1b with significantly lower affinity than it does to *dinBox*1. In contrast, when EMSA was performed with a probe mutated at *dinBox*1b, the faster-migrating complex was still observed but not the slower-migrating form (Figure 2A, lanes 14–20). A doubly mutated probe could not be shifted by His_6_-LexA (Figure 2A, lanes 21–26). The mutant probes, which in each case eliminated respective LexA binding, were designed using the known consensus sequence for LexA binding in other bacterial species, leading us to conclude that *B. thuringiensis* LexA makes similar important and specific protein:DNA contacts. We did not observe a third, slower-migrating species that would indicate LexA binding simultaneously to both *dinBox* sites. Either the close proximity of *dinBox*1 and *dinBox*1b prevents dual binding or this complex was not observed in EMSA for unknown reasons.

Surface plasmon resonance (SPR) analysis validated our EMSA results. His_6_–LexA interacted with the wild-type operator targets in a concentration-dependent manner (Figure 2B). Association of His_6_-LexA with an immobilized DNA fragment carrying either single or double nucleotide changes in *dinBox*1 was still detectable but was unstable, indicating that *dinBox*1b is a low-information-content-site. In contrast, a strong and very stable LexA-DNA interaction was observed when the DNA fragment contained a mutated *dinBox*1b operator (Figure 2B), indicating that *dinBox*1 is indeed a high-affinity binding site. These data show that LexA is capable of binding independently to the potentially overlapping sites *dinBox*1 and *dinBox*1b in the *P1* promoter region and that there are noticeable differences in the affinity and stability of binding at each site.

Since *dinBox*1b can function as a LexA-binding site, we assessed its role in regulating gene expression from *P1* by studying a *lacZ* fusion with a nucleotide substitution in the *dinBox*1b half-site (Figure 1A) in the cured and lysogenic hosts (Figure 1B, right panel). LacZ assays showed that expression levels from a *dinBox*1b mutant were higher in the cured and lysogenic *lexA*^+^ strains compared with the wild-type fusion, indicating that LexA repression is slightly lifted when *dinBox*1b is mutated but SOS-induction levels are maintained. We conclude that *dinBox*1b plays a minor but detectable role in *P1* transcription in vivo.

#### LexA protects a sequence encompassing *dinBox*1 and *dinBox*1b

In order to physically define the LexA binding sites in the *P1* promoter region, DNase I protection assays were performed using radiolabeled wild-type or mutant DNA fragments (sequences are shown in Figure 1A). Figure 2C shows protection data obtained with the wild-type promoter sequence as well as derivatives mutated in either *dinBox*1, *dinBox*1b or in both sites simultaneously. As can be seen for the wild-type probe (lanes 1–4), the location and extent of protection with increasing concentrations of His_6_-LexA are consistent with His_6_–LexA binding to both *dinBox*1 and *dinBox*1b. Full occupancy of the *dinBox*1 site occurred at 320 nM His_6_-LexA, whereas at the same LexA concentration, *dinBox*1b is only partially protected (Figure 2C, lane 4). In addition, DNase I hypersensitive sites were detected at the boundaries of (positions +26 to +27 and +43) and within the *dinBox*1b site (positions +33 to +34, shown with arrows in Figure 2C), indicating that local distortions or bends of the DNA result from His_6_-LexA binding. Curiously, the extent of His_6_–LexA binding to *dinBox*1 differs from that observed for *dinBox*1b. Considering that the *Bacillus* LexA dimer binds to a 14-bp sequence, the expected length of a footprint would be 18–20 bp. This is the case for *dinBox*1 but not for *dinBox*1b, for which the protected sequence corresponds to ∼14 bp, raising the possibility that His_6_-LexA does not bind to *dinBox*1b DNA in the same manner. Alternatively, the *dinBox*1 and *dinBox*1b sites overlap and prevent simultaneous binding of two LexA dimers.

Compared to a wild-type probe, His_6_-LexA binding to a probe mutated in *dinBox*1 (CGAACaagc**T**TTTTataagtGTTCG, mutation shown in bold) is considerably reduced (Figure 2C, compare lanes 5–8 to lanes 1–4). Using the same probe, His_6_-LexA binding to *dinBox*1b is unchanged, except for the loss of one hypersensitive site within *dinBox*1b (at positions +33 to +34) that is associated with LexA binding to *dinBox*1. A point mutation in *dinBox*1b (CGAACaagcGTTTTataagtGTTC**A**) significantly interferes with His_6_-LexA binding to the *dinBox*1b site while binding to *dinBox*1 remains unchanged (Figure 2C, compare lanes 9–12 to lanes 1–4). However, the hypersensitive site within *dinBox*1b (at positions +33 to +34) is restored, consistent with this hypersensitive site being associated with LexA binding to *dinBox*1. A *dinBox*1–*dinBox*1b double mutant (CGAACaagc**T**TTTTataagtGTTC**A**) showed no specific interaction with His_6_–LexA (Figure 2C, lanes 13–16).

Taken together with the EMSA results (Figure 2A), the DNase I footprint analysis leads us to conclude that His_6_-LexA binds independently to *dinBox*1 and *dinBox*1b and that His_6_-LexA binding is associated with DNA distortions.

#### Gp7 interacts with LexA at *dinBox*1 and *dinBox*1b

Since a *P1-lacZ* fusion is more strongly repressed in a lysogen than in the cured host (Figure 1B, left panel), we suspected that, in addition to LexA, phage factors might also be involved in the regulation of *P1*. In a previous study, an additional class of GIL01 *clear-plaque* mutants had been isolated in ORF7, coding for a small protein of unknown function (17). Complementation studies showed that ORF7 was essential for GIL01 to enter the lysogenic state. To explore the possible role of gp7 in GIL01 regulation, ORF7 was over-expressed in *E. coli* as an N-terminal 6xHis fusion and used in EMSA experiments. Although His_6_–gp7 did not bind to a DNA probe encompassing the *P1* region, His_6_-gp7 gave rise to a slower-migrating novel form when added to reactions together with His_6_-LexA (Figure 3). This interaction persisted when the DNA probes were mutated in *dinBox*1 (lanes 5–8) or *dinBox*1b (lanes 9–12), although the intensity of the interaction was diminished with a *dinBox*1-mutated probe and was weakest with a probe mutated at the *dinBox*1b site. These results suggest that gp7 can form a ternary complex with LexA bound at *dinBox*1 or at *dinBox*1b.

#### Gp7 increases the apparent binding capacity of LexA for *dinBox*1 and *dinBox*1b

**Figure 4.**
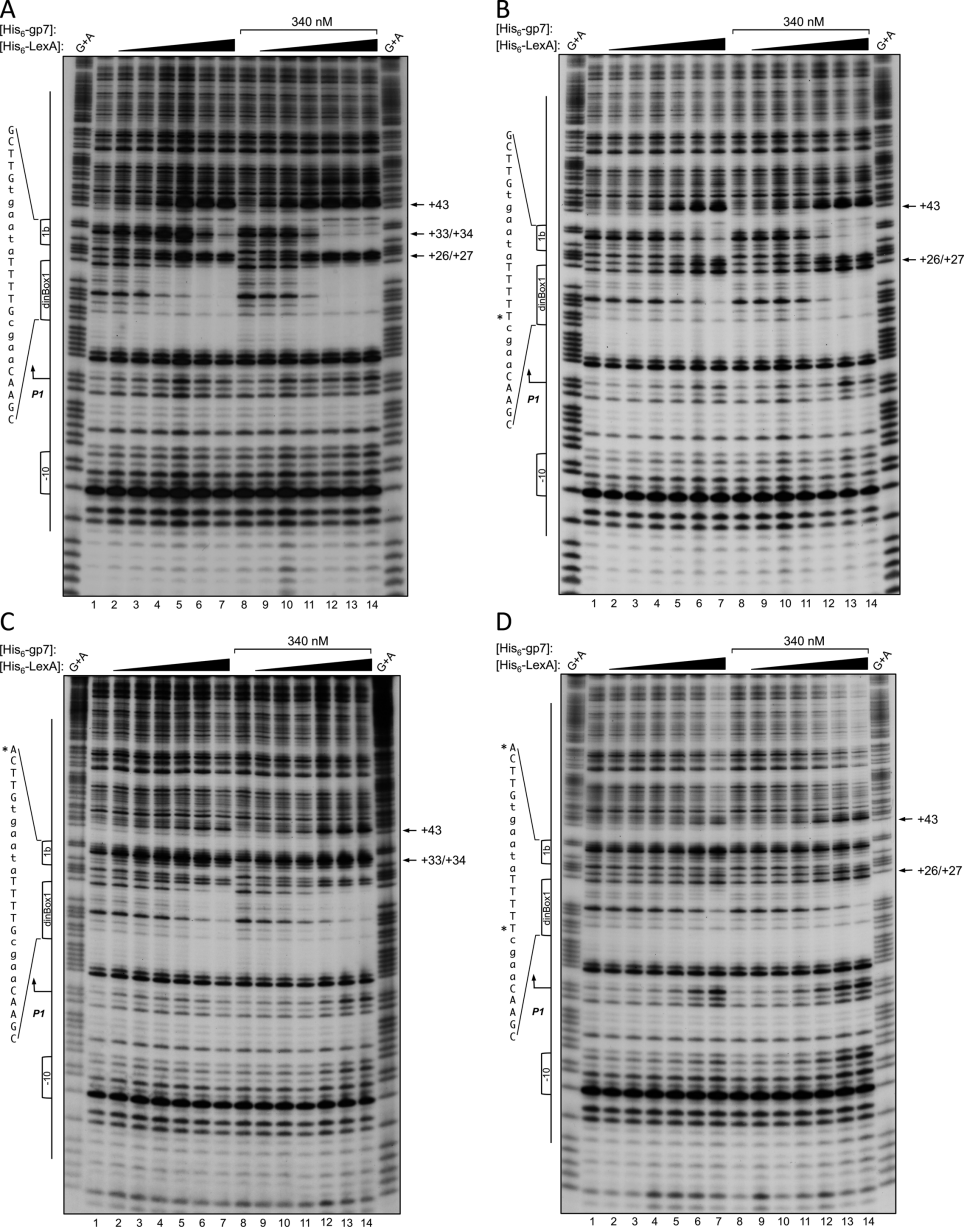
DNase I footprint analysis showing enhanced LexA binding to *dinBox*1 and *dinBox*1b in the presence of gp7. DNA fragments extending from −39 to +176 relative to the start of *P1* transcription (see Figure 1A) were ^32^P-labeled at the 5′ end of the top strand, incubated with His_6_-LexA or His_6_–LexA plus His_6_–gp7, and digested with DNase I. Cleavage products were separated on an 8% sequencing gel. G+A marker lanes contain the respective probes chemically cleaved at guanosine and adenosine residues. Lane 1 is probe DNA without added protein. His_6_–LexA concentrations in lanes 2–7 and 9–14 are 10, 20, 40, 80, 160 and 320 nM. In lanes 8–14, His_6_–LexA was pre-incubated with the DNA probe prior to addition of 340 nM His_6_–gp7. SOS box sequences are indicated in the left margin and the nucleotide positions that were substituted to generate the *dinBox*1 and *dinBox*1b mutated probes are indicated by an asterisk. The *P1* transcription start site is denoted with an angled arrow and the -10 sequence is indicated. Arrows in the right margin denote DNase I hypersensitivity sites. His_6_–LexA and His_6_–gp7 binding was studied to wild-type (**A**), mutated *dinBox*1 (**B**), mutated *dinBox*1b (**C**) and mutated *dinBox*1-*dinBox*1b (**D**) probes. The G+A marker in (B) corresponds to the wild-type probe and not the *dinBox*1 mutant probe.

In DNase I footprints (Figure 4), His_6_-gp7 alone did not protect a DNA probe encompassing promoter *P1* (Figure 4A, lane 8). However, when His_6_–gp7 was added to the DNA probe along with His_6_–LexA, His_6_–gp7 clearly stabilized LexA binding to *dinBox*1 and *dinBox*1b (Figure 4A, lanes 9–14). In particular, His_6_-gp7 greatly increased apparent binding affinity of His_6_–LexA to *dinBox*1b. Binding to *dinBox*1 improved only slightly upon addition of His_6_-gp7. Overall, the presence of His_6_–gp7 resulted in His_6_-LexA occupying *dinBox*1 and *dinBox*1b at markedly lower LexA concentrations (Figure 4A, lanes 8–14). We extended these observations by testing the ability of His_6_–gp7 to promote His_6_-LexA binding to DNA probes mutated in *dinBox*1 and/or *dinBox*1b. DNase I footprint analysis showed that when *dinBox*1 carried a single nucleotide mutation that does not entirely abolish His_6_–LexA binding, His_6_–gp7 was still seen to enhance His_6_–LexA binding to *dinBox*1 by approximately two-fold (Figure 4B). Similar to what we observed with the wild-type probe, addition of His_6_–gp7 resulted in His_6_–LexA occupying both sites at significantly lower LexA concentrations. In contrast, when the probe is mutated in *dinBox*1b, the effect of His_6_–gp7 on LexA binding at *dinBox*1b site is barely detectable (Figure 4C). A doubly mutant probe is no longer bound by His_6_–LexA and the enhancing effect of His_6_–gp7 is only detectable at the highest His_6_–LexA concentrations (Figure 4D, lanes 13 and 14). Again, we did not observe DNase I protections that would indicate gp7 making direct contacts with *P1* promoter DNA. Altogether, these results suggest that His_6_-gp7 does not make direct DNA contacts but instead cooperates with His_6_-LexA to enhance DNA binding. This could be the case if gp7 favored a LexA conformation that is better suited for DNA binding.

**Figure 5.**
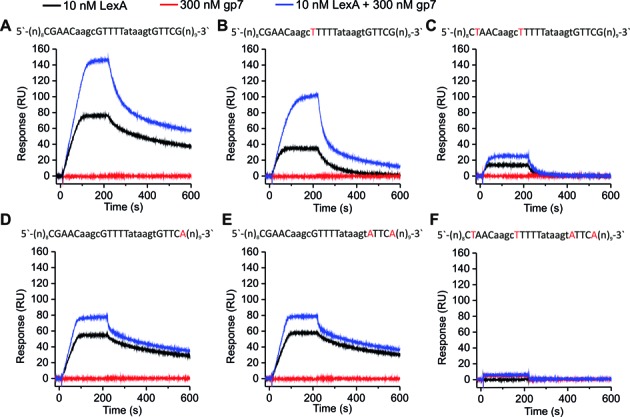
SPR analysis of the gp7 interaction with LexA bound to the *dinBox* sites. (**A–F**) SPR sensorgrams showing the interaction of His_6_–LexA and His_6_–gp7, alone or combined, with a DNA fragment carrying *dinBox*1 and *dinBox*1b. Proteins were injected across each chip-immobilized DNA fragment (∼60 RU) for 210 s at a flow rate of 100 μl/min. Protein concentrations are denoted above the sensorgrams and operator sequences are presented above each graph with mutated nucleotides shown in red. The experiments were performed in triplicate and representative sensorgrams are shown (See also Supplementary Figures S2, S3 and S4).

To provide further insights into this interaction, we used SPR to measure the gp7 effect on LexA–DNA binding. Purified His_6_–gp7, at a concentration of 300 nM, did not appear to directly interact with any of the DNA fragments used in SPR analysis (Figure 5). However, it strongly enhanced His_6_-LexA DNA binding capacity for the streptavidine (SA) chip-immobilized DNA fragment carrying *dinBox*1 and *dinBox*1b (Figure 5A). An increased His_6_-gp7 effect on LexA binding capacity was also observed when His_6_-gp7 was added together with His_6_-LexA to an immobilized DNA fragment harboring a point mutation in *dinBox*1 (Figure 5B). In contrast, His_6_–gp7 had markedly smaller effect on His_6_-LexA binding when *dinBox*1 was eliminated due to the change of two conserved nucleotides in *dinBox*1 (Figure 5C). When either single or double mutations were introduced into *dinBox*1b, addition of His_6_–gp7 could not restore optimal LexA binding, as observed for the wild-type fragment in presence of His_6_–gp7 (Figure 5D and E). We determined that His_6_–gp7, in the concentration range of 75–300 nM, had similar effects on His_6_–LexA binding and therefore, a His_6_–gp7 concentration of 300 nM was used in all subsequent SPR experiments (Supplementary Figure S2). The SPR data showed that His_6_–gp7 enhances LexA binding to both *dinBox* sites but not to the non-specific DNA (Figure 5F). We hypothesize that phage factor gp7 modulates LexA DNA binding affinity by stabilizing a specific repressor conformation (21). We also measured the effect of His_6_–gp7 on His_6_–LexA binding to the known SOS box upstream of *B. thuringiensis lexA*, or its mutant variant containing three nucleotide substitutions. LexA interacted with the wild-type operator in a concentration-dependent manner and the complexes were highly stable. Interaction with the mutated DNA probe was detectable but the complexes were markedly unstable (Supplementary Figure S3). SPR analysis confirmed that His_6_–gp7 enhances LexA binding to both DNA fragments.

To further show that gp7 stabilizes LexA binding to DNA targets, we pre-incubated His_6_–LexA and His_6_–gp7 and then injected them over the chip-immobilized DNA fragment harboring wild-type *dinBox* sites and followed the dissociation of the resulting nucleoprotein complexes. Data showed that subsequent injection of additional His_6_–gp7 prevented His_6_–LexA from dissociating off the DNA fragment (Supplementary Figure S4A). Collectively, our results strongly suggest that gp7 interacts with LexA to enhance its DNA binding affinity at a variety of target sites.

#### Gp7 interacts with free LexA repressor

**Figure 6.**
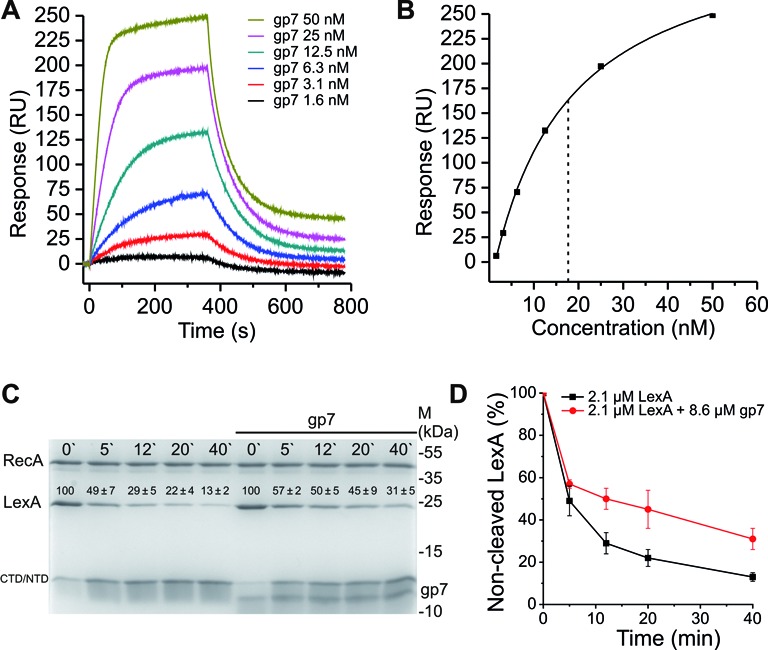
Gp7 reduces the rate of LexA auto-proteolysis in the presence of activated RecA. (**A**) SPR sensorgram of His_6_–gp7 interaction with immobilized His_6_–LexA. His_6_–gp7 was injected in a concentration range of 1.6-50 nM and flowed across the chip-immobilized LexA (∼2000 RU) for 360 s at a rate of 30 μl/min. The dissociation phase was followed for 420 s. (**B**) Determination of the apparent equilibrium dissociation constant (*K*_D_) from the plot of response as a function of His_6_–gp7 concentration injected across His_6_–LexA. For this analysis, we assumed a stoichiometry of one gp7 monomer per LexA monomer. The dotted line indicates an apparent *K*_D_ for His_6_–gp7 binding to His_6_–LexA of approximately 17.7 nM. (**C**) Time course of His_6_–LexA auto-proteolysis in the presence of RecA-ATP filaments. LexA was pre-incubated with gp7 in a molar ratio of ∼1:4 (LexA monomer:gp7 monomer) and added to pre-formed RecA-ATP filaments (2 μM). The inhibition of LexA auto-proteolysis by gp7 was monitored by SDS-PAGE. Quantitation of each auto-cleavage reaction is shown above the respective band as the ratio (%) of the protein density value of the initial sample (0 min) relative to the density value obtained from the proteins after 5, 12, 20 or 40 min after addition of the active RecA filament, together with the standard error of the mean. Intact LexA monomer (LexA), C-teminal or N-terminal LexA fragments (CTD/NTD) and RecA protein are marked accordingly. Experiments were performed in duplicate and a representative gel is shown. (See also Supplementary Figure S5.) (**D**) Graphical representation of intact LexA resulting from incubation with activated RecA, with or without gp7, as presented in panel (C).

The direct interaction of LexA with another protein has not been described yet. Thus, to investigate the possibility of a direct interaction between LexA and gp7, we injected the purified His_6_-gp7 in different concentrations across the chip-immobilized *B. thuringiensis* LexA repressor, in the absence of any DNA. SPR analysis shows that His_6_–gp7 and His_6_–LexA interacted with high affinity, exhibiting an apparent equilibrium dissociation constant (*K*_D_) of 17.7 nM for the predicted stoichiometry of one gp7 monomer per LexA monomer (Figure 6A and B). The specificity of the interaction was further confirmed by the absence of interaction between LexA and another phage-borne protein, predicted DNA binding protein gp1 (Supplementary Figure S4B). When the experiment was reversed and His_6_–gp7 was immobilized onto the surface of the CM5 chip, no interaction with free His_6_–LexA was detected, which suggests that the entire surface of His_6_–gp7 must be exposed for the interaction to occur (Supplementary Figure S4B). We conclude that gp7 directly interacts with the LexA repressor, likely changing its conformation in a way that enhances LexA binding affinity for its target DNA.

Since His_6_–gp7 interacts with His_6_-LexA, we reasoned that such an interaction could interfere with RecA-mediated LexA auto-cleavage. To investigate this, activated RecA filaments were pre-formed with ATP and ssDNA, and added to His_6_-LexA that had been pre-incubated with His_6_–gp7 at approximately one LexA dimer per eight gp7 monomers. Proteolytic cleavage of His_6_–LexA was monitored by SDS-PAGE. These results showed that His_6_-gp7 moderately inhibits the rate of RecA-catalyzed LexA auto-cleavage, compared to the rate of LexA inactivation when tested in the absence of His_6_–gp7 (Figure 6C and D). No His_6_–LexA cleavage was visible in the presence of inactive RecA (without addition of ATP and ssDNA) (Supplementary Figure S5). Our data suggest that viral protein gp7 can directly modulate the bacterial SOS response *via* a previously undescribed mechanism, by direct protein interaction with LexA repressor.

#### Gp7 represses promoter *P1* and prevents its SOS induction in vivo

**Figure 7.**
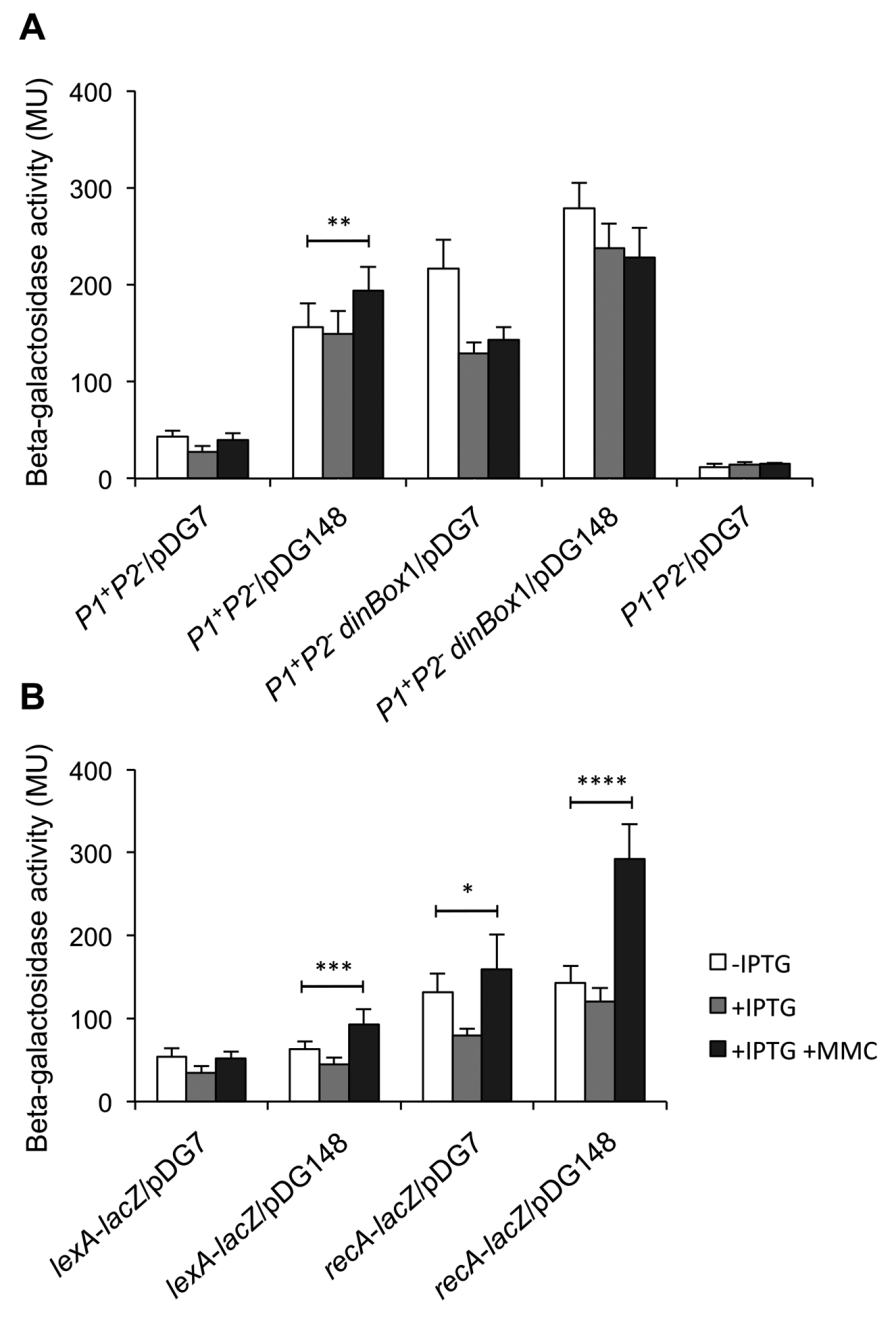
Expression of gp7 in *B. thuringiensis* enhances LexA-mediated repression at promoter *P1* and prevents SOS gene induction. (**A**) Beta-galactosidase activity from a *P1*^+^*P2*^−^-*lacZ* fusion was measured in the absence (−IPTG) or presence (+IPTG) of gp7 in *B. thuringiensis* GBJ002. After 1 h of IPTG (0.1 mM) induction, the cells were treated with MMC50 for 1 h in order to elicit the SOS response. pDG148 is the insert-free vector used as control and pDGP7 is the pDG148 vector expressing gp7. Note that the *spac* promoter is leaky in *B. thuringiensis* (*P1*^+^*P2*^−^/pDG7; −IPTG). Upon induction of gp7 expression (+IPTG), *P1*^+^*P2*^−^ repression by gp7 is similar to that observed in the lysogen (Figure 1B, left panel). Data represent mean ± S.D. from three independent replicates, and statistical significance was by two-tailed *t* test (*P* values: *, <0.05; **, <0.005; ***, <0.0005; and ****, <10^−6^). (**B**) Same experiment as in (A) using *lexA* and *recA* promoter fusions.

In order to assess the role of gp7 in *P1* transcription in vivo, ORF7 was cloned downstream of the inducible *spac* promoter in pDG148, a vector capable of replicating in *Bacillus* (17). We determined the effect of gp7 expression on a *P1*^+^*P2*^−^-*lacZ* fusion in the GIL01 host, *B. thuringiensis*. As can be seen in Figure 7A, the *spac* promoter is leaky and strong repression of *P1*^+^*P2*^−^ was observed in the absence of IPTG induction. Previous studies indeed showed that the *spac* promoter on multicopy vector pDG148 directs basal levels of transcription in the absence of inducer (23,24). Induction of gp7 expression by addition of IPTG (0.1 mM) to growing cells resulted in increased repression of *P1*. When mitomycin C was added to the cultures expressing gp7 in presence of the *P1*^+^*P2*^−^-*lacZ* fusion, *P1* repression was not lifted as we observed for the same fusion in a normal lysogen host (Figure 1B, left panel). This result shows that gp7 over-expression strongly enhances repression of *P1* and also prevents induction of *P1* by SOS, likely through its interaction with host LexA.

To confirm that gp7 repression of *P1* is LexA-dependent, we analyzed the effect of gp7 expression on a *P1*^+^*P2*^−^ -*lacZ* fusion mutated in *dinBox*1 (Figure 7A). We know from EMSA and DNase I footprint experiments that His_6_–LexA affinity for a mutated *dinBox*1 site is considerably weakened and that addition of His_6_–gp7 only slightly improves His_6_-LexA binding. *In vivo*, gp7 has a modest effect on a *P1*^+^*P2*^−^ *dinBox*1mut-*lacZ* fusion (similar to the repression levels observed with a same fusion in a GIL01-lysogen, Figure 1B), indicating that binding of LexA to *dinBox*1 is required to observe gp7-mediated repression. Taken together, these results show that gp7 prevents SOS-activation of *P1* by acting at the level of LexA.

In order to determine if the gp7-mediated repression extends to other cellular SOS genes, similar experiments were carried out using *lexA* and *recA* promoter fusions to *lacZ*. As shown in Figure 7B, over-expression of gp7 in *B. thuringiensis* did not noticeably enhance LexA-mediated repression of *lexA* and *recA*, but gp7 expression did inhibit their SOS induction. These results confirmed that gp7 can act as an accessory factor of host LexA and thereby, inhibit the induction of the SOS response.

## DISCUSSION

Our previous results suggested a direct role for LexA in regulating transcription from the GIL01 tandem promoters *P1* and *P2* (17). Here we show that LexA specifically binds to and regulates promoter *P1* and is assisted by GIL01-encoded gp7 in repressing transcription. LexA binds to two sites downstream of promoter *P1* but does not efficiently repress *P1* transcription unless gp7 is also provided. We found that gp7 enhances LexA binding to operator DNA by forming stable complexes with free or DNA-bound LexA through a direct physical interaction. Gp7 does not appear to contain a known DNA binding domain and none of our data suggest that gp7 makes direct contact with the DNA. Most importantly, expression of gp7 inhibits induction of the host SOS response and, in vitro, impairs RecA-stimulated proteolysis of LexA. This study identifies for the first time a factor that interacts with the SOS repressor, LexA, to increase its affinity for DNA and inhibit its auto-cleavage.

### Host LexA binds to a noncanonical site in phage GIL01

Our EMSA and footprinting studies show that LexA binds to two distinct sites located downstream of the *P1* transcription start. While *dinBox*1 nearly matches the LexA binding motif found in Gram-positive bacteria (25), *dinBox*1b only displays a single recognizable half-site. A mutation in *dinBox*1 results in the loss of LexA regulation and commits GIL01 to exclusively carry out the lytic cycle. In contrast, the *dinBox*1b mutation has little effect on LexA regulation and no lytic mutants were isolated in *dinBox*1b (17). In agreement with the relative importance of both sites in vivo, our mobility shift assays and SPR analysis show that LexA has high affinity for *dinBox*1 while binding to the *dinBox*1b half-site is noticeably unstable. In *E. coli*, LexA primarily exists as a dimer that recognizes a DNA sequence of inverted symmetry (26,27). The LexA dimer was shown to be able to bind to operator half-sites, but it did so with considerably lower affinity (28). This could also be the case for *B. thuringiensis* LexA repressor and would explain the poor affinity for *dinBox*1b as well as a shorter footprint at *dinBox*1b (Figure 2C). One LexA monomer might bind the cognate half-site specifically, while the other monomer binds operator DNA non-specifically.

One intriguing observation in EMSA experiments is the absence of an apparent third shifted form representing doubly bound probe, with LexA bound simultaneously to *dinBox*1 and *dinBox*1b. It is possible that LexA binding to *dinBox*1b generates DNA distortions that are not compatible with simultaneous LexA occupation of both sites. We also considered the possibility of cooperative binding at *dinBox*1 and *dinBox*1b. Considering that *dinBox*1b is a weak-affinity site, one might expect LexA binding to the higher affinity site, *dinBox*1, to provide the cooperative energy that assists LexA binding to *dinBox*1b. However, our in vitro DNA binding results suggest that there is no cooperativity between bound LexA dimers and even questions the possibility of simultaneous LexA binding to both sites.

### LexA binding is assisted by phage-borne gp7

A previous study in *E. coli*, using chromatin immunoprecipitation coupled with high-density microarrays, reported LexA binding to noncanonical SOS box sites. Since LexA binding appeared to be overall restricted to biologically relevant sites, the authors hypothesized that LexA is likely to cooperate with an accessory factor to enable binding at these poorly conserved sites (15). We identified phage-borne gp7 as a factor cooperating with *B. thuringiensis* LexA. In our experiments, gp7 did not appear to interact directly with the DNA but formed a ternary complex with LexA and its cognate binding sites. Gp7 interacting with LexA, but without contacting the DNA, would constitute a unique example of a transcription accessory factor in bacteria. Transcription factors cooperating to regulate transcription are typically DNA binding proteins that prevent or facilitate RNAP-promoter binding by direct interaction or indirectly, by modifying the DNA topology. One well-documented example is the cooperative interaction between the global transcription regulator cAMP receptor protein (CRP) and CytR in *E. coli* (29). Both are DNA binding proteins but CytR cannot stably bind to promoter DNA by itself: efficient DNA binding of CytR relies on co-binding with CRP-cAMP. The binding of one protein increases the affinity of the other protein for an adjacent binding site through specific protein:DNA and protein:protein interactions. Similarly, CRP and MelR need to cooperate in order to increase DNA occupancy by MelR and efficiently activate downstream gene transcription (30). In other systems, such as the *B. subtilis* lytic phage phi29, transcription regulator p4 interacts with histone-like protein p6 to form a hairpin structure that is instrumental in the early-to-late gene transcription switch (31).

Here, gp7 augments the binding affinity of LexA for conserved sites but appears to assist even more in LexA binding to poorly conserved sites such as *dinBox*1b. As a result, there is much tighter repression of promoter activity as well as impaired SOS induction of the LexA/gp7-regulated promoter. It is noteworthy that gp7 influences LexA binding to neighboring sites, such as at promoter *P1*, as well as to isolated LexA operators, such as those found upstream of *recA* and *lexA*, suggesting that the promoter-SOS box configuration, i.e. the number and distribution of the SOS boxes, is not a determining factor for gp7 activity. Since we provide evidence of a direct LexA-gp7 physical interaction, the next question we asked is how gp7 influences LexA function. Since LexA essentially exists as a dimer in the cell, the function of gp7 is not to improve dimer formation but to favor DNA binding by affecting the conformation of LexA. LexA monomers dimerize by the carboxyterminal domain (CTD) (32) and bind to specific DNA sequences via a helix-turn-helix in the amino-terminal domain (NTD) (33). Previous studies showed that a reorientation of the LexA DNA binding NTD with respect to the CTD was necessary for stable and specific operator binding (34,35). It is possible that gp7 somehow favors this reorientation and by doing so, induces the non-cleavable conformation of LexA. Gp7 could influence LexA binding by interacting with its N-terminus or the hinge that connects the NTD to the CTD (36). Likewise, LexA auto-cleavage could be prevented by gp7 directly interfering with the RecA-ssDNA interaction. The current structural model in *E. coli* shows that activated RecA filament interacts with whole LexA (37), offering many LexA-gp7 interaction possibilities that would impede access to RecA. More studies, including elucidating the structure of the LexA-gp7 complex, are required in order to shed light on the nature of this interaction.

### The dynamics of the SOS response

LexA regulates the lytic cycle of several phages, usually by repressing the expression of anti-immunity factors (12–14) but rarely by directly controlling the expression of phage propagation genes (11). Here, LexA directly regulates the lytic cycle of GIL01 by binding to both conserved and poorly conserved operators with the help of a phage-borne accessory factor. Gp7 not only stabilizes LexA binding but also inhibits the RecA-catalyzed auto-proteolysis of LexA, a step that is critically important to initiate lytic growth of GIL01. GIL01, unlike phage lambda, does not code for its own SOS-sensitive repressor but uses host LexA to control lytic and lysogenic gene expression (17). In lambda, CI cleavage in response to genomic stress occurs at a slower rate than LexA cleavage (38), possibly in a bid to ensure that the phage cycle is only induced once the cell is no longer able to address DNA damage. If GIL01 lytic genes were regulated by LexA alone, stable lysogeny would only be achieved through high LexA affinity for its sites or an important number of sites, such that the phage is only induced in response to a significant SOS signal. By expressing gp7, GIL01 might ensure that the lytic cycle is stably repressed in normal growth conditions and induced only in response to a persistent DNA damage signal. The effect of gp7 on the host cellular response to genomic stress, on the other hand, would have considerable impact on the host's cell biology. By delaying or impairing the SOS response, gp7 would modulate the response that senses DNA damage and would therefore affect cell repair and survival. Since we observed that gp7 was strongly expressed during SOS induction (Supplementary Figure S6), a delay or inhibition of the SOS response is very likely to occur. By expressing gp7, GIL01 determines the timing and extent of the SOS response, playing an important role in cell fate.

We only found gp7 homologues in tectivirus genomes that are related to GIL01 (Supplementary Figure S7). Therefore, it seems that peptides with a similar function are rare and only found in phage genomes. This fits within the view of bacteriophages coding for functions that coopt the host into following the phage developmental program. Other phages code for their own sigma factors and DNA metabolism proteins (T4) (39), addiction systems (P1) (40), quorum sensing genes (41), all with the purpose of controlling the host metabolic machinery for their own benefit. This study describes a phage-encoded protein that specifically interferes with the host SOS DNA repair pathway.

Since LexA continues to be the subject of numerous studies because of its important role in the cell, and the insights it provides into regulatory circuits of higher organisms, a fuller description of our findings will have important consequences for understanding the dynamics of the SOS response. Essentially, gp7 is a promising inhibitor of the SOS response by virtue of its strong interaction with *B. thuringiensis* LexA repressor.

## SUPPLEMENTARY DATA

Supplementary Data are available at NAR Online.

SUPPLEMENTARY DATA
