# Supplementary material for: Bacteriophage GIL01 gp7 interacts with host LexA repressor to enhance DNA binding and inhibit RecA-mediated auto-cleavage
Source: Nucleic Acids Res. 2015 Jul 2;43(15):7315–29. doi: 10.1093/nar/gkv634 (PMC4551915; doi:10.1093/nar/gkv634)
Supplement: SUPPLEMENTARY DATA [file supp_43_15_7315__index.html]

Bacteriophage GIL01 gp7 interacts with host LexA repressor to enhance DNA binding and inhibit RecA-mediated auto-cleavage — Bacteriophage GIL01 gp7 interacts with host LexA repressor to enhance DNA binding and inhibit RecA-mediated auto-cleavage — Bacteriophage GIL01 gp7 interacts with host LexA repressor to enhance DNA binding and inhibit RecA-mediated auto-cleavage — SUPPLEMENTARY DATA 

# Bacteriophage GIL01 gp7 interacts with host LexA repressor to enhance DNA binding and inhibit RecA-mediated auto-cleavage

## SUPPLEMENTARY DATA

- SUPPLEMENTARY DATA
