# Supplementary material for: Bacteriophage GIL01 gp7 interacts with host LexA repressor to enhance DNA binding and inhibit RecA-mediated auto-cleavage
Source: Nucleic Acids Res. 2015 Jul 2;43(15):7315–29. doi: 10.1093/nar/gkv634 (PMC4551915; doi:10.1093/nar/gkv634)
Supplement: SUPPLEMENTARY DATA [file supp_gkv634_nar-01282-m-2015-File002_revised.pdf]

Supplementary Data

Bacteriophage GIL01 gp7 interacts with host LexA repressor to enhance DNA binding and inhibit RecA-mediated auto-cleavage.

Nadine Fornelos*, Matej Butala, Vesna Hodnik, Gregor Anderluh, Jaana K. Bamford, and Margarita Salas

* To whom correspondence should be addressed. Email: nadine.fornelos@jyu.fi

Supplementary Method

RNA extraction and quantitative RT-PCR

Total RNA was extracted from strains GBJ002 and GBJ338, grown under normal and SOS-inducing conditions, using the RiboPure-Bacteria Kit (Ambion). Three biological replicates per strain and growth condition were made. To eliminate DNA contamination, the extracts were treated with DNase I (Ambion). Extracted RNA was quantified on a Bioanalyzer 2100 with the RNA 6000 Nano LabChip kit (Agilent). The absence of contaminating genomic DNA in RNA preparations was verified using RNA as a template in PCR assays. Oligonucleotides for real-time PCR (Table S1) were designed using the NCBI Primer-BLAST tool, which included a BLAST analysis against *B. cereus G9842* genome sequence for specificity confidence. 40 ng of total RNA was reverse-transcribed into cDNA in a 20 µl reaction mixture using the iScript cDNA synthesis kit (Bio-Rad). The cDNA levels were then analyzed using the CFX96 real-time PCR system from Bio-Rad. Each sample was run in triplicate in a 96-well plate with the iQ SYBR Green Supermix (Bio-Rad) in a total volume of 25 µl. Non-template controls (NTC) were also included in triplicate for each primer pair. The thermocycling program consisted of one hold at 95°C for 3 min, followed by 40 cycles of 10 s at 95°C, 20 s at 60°C and 20 s at 72°C. PCR specificity, contamination and the absence of primer dimers was deduced from melting curve analysis in CFX Manager (Bio-Rad) and by inspecting the length of the PCR product in agarose gel electrophoresis. Quantification cycle values (Cq) were extracted to GenEx (MultiD Analyses) where they were normalized with inter-plate calibrators to adjust for run-to-run differences, corrected for reaction efficiency, averaged for one same strain and condition, and normalized to reference genes *gyrB* and *tufA*.

Supplementary Reference

1. Waterhouse, A.M., Procter, J.B., Martin, D.M., Clamp, M. and Barton, G.J. (2009) Jalview Version 2--a multiple sequence alignment editor and analysis workbench. *Bioinformatics*, **25**, 1189-1191.

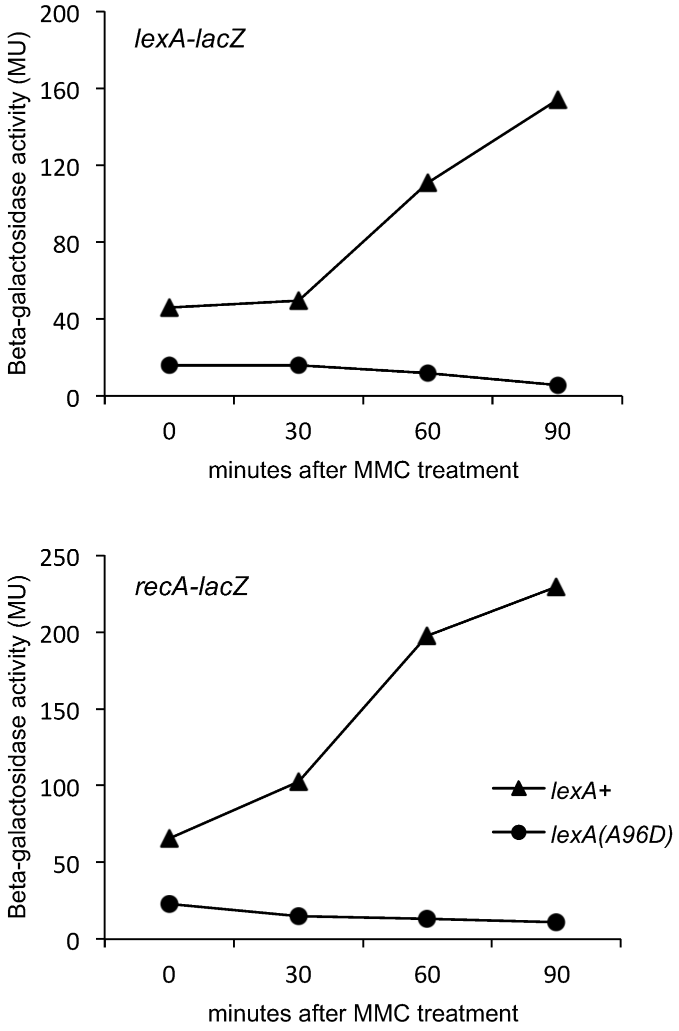

Supplementary Figure S1. SOS induction of the host *lexA* and *recA* promoters only occurs in a *lexA*$^+$ host.

The *lexA* and *recA* promoters, each encompassing a single SOS box, were cloned upstream of the promoterless *lacZ* gene in the shuttle vector pHT304-18Z. Beta-galactosidase activity was measured in *B. thuringiensis lexA*$^+$ and non-inducible *lexA(A96D)* hosts at the indicated times after mitomycin C treatment (MMC 0.05 µg/ml) of growing cultures.

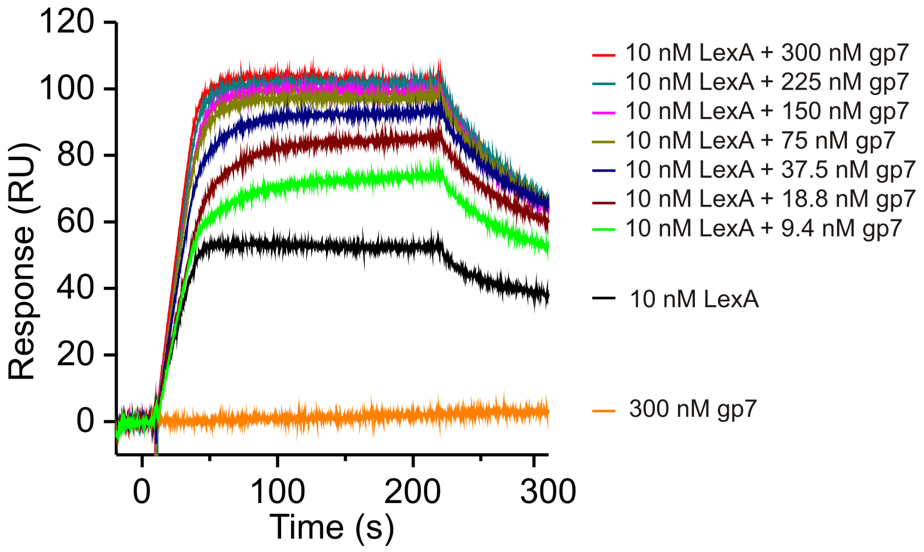

Supplementary Figure S2. SPR analysis of the gp7 interaction with LexA bound to the *dinBox* sites.

A DNA fragment containing the *dinBox*1 and *dinBox*1b sites was immobilized (30 RU) on the surface of the streptavidin sensor chip. Purified His_6-LexA or His_6-LexA plus His_6-gp7 were injected at the indicated concentrations across the 42 bp DNA fragment for 210 s at a flow rate of 100 µl/min and dissociation was followed for 100 s. The experiment was performed in duplicate and a representative graph is shown. RU, response units.

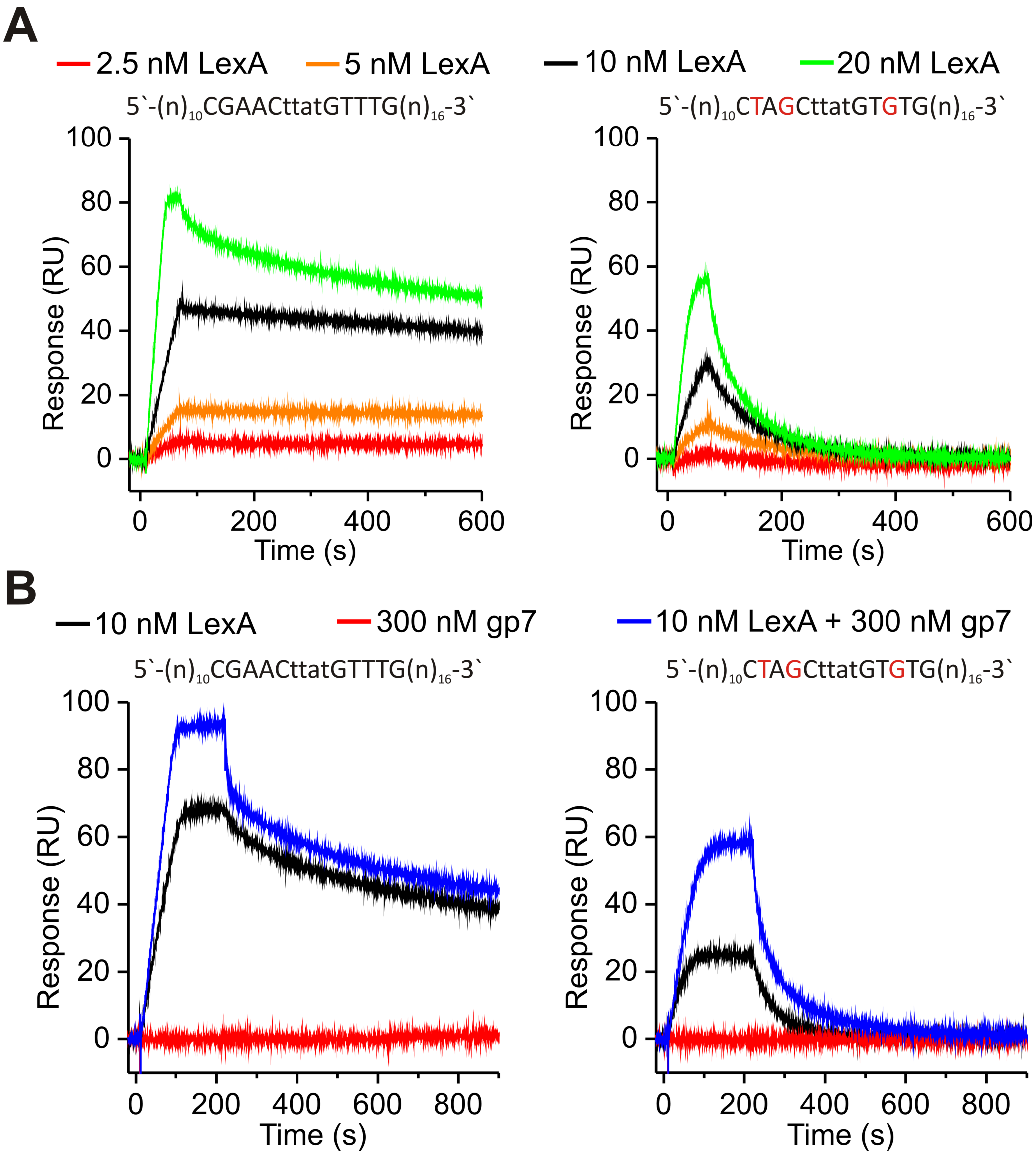

Supplementary Figure S3. Interaction of LexA and LexA pre-incubated with gp7 with the *lexA* gene promoter region.

(A) His_6-LexA in a concentration range of 2.5–20 nM was injected for 60 s at a flow rate of 100 µl/min across a chip-immobilized DNA fragment harboring the regulatory region of the *lexA* gene (~50 RU).

(B) SPR sensorgrams showing the interaction of His_6-LexA and His_6-gp7, alone or combined, with intact or mutated *lexA* gene operator DNA. Proteins were injected across chip-immobilized DNA fragments (~50 RU) for 210 s at a flow rate of 100 µl/min. Protein concentrations are denoted above the sensorgrams and operator sequences are presented above each graph with mutated nucleotides shown in red. Experiments in (A) and (B) were performed in triplicate and representative sensorgrams are shown.

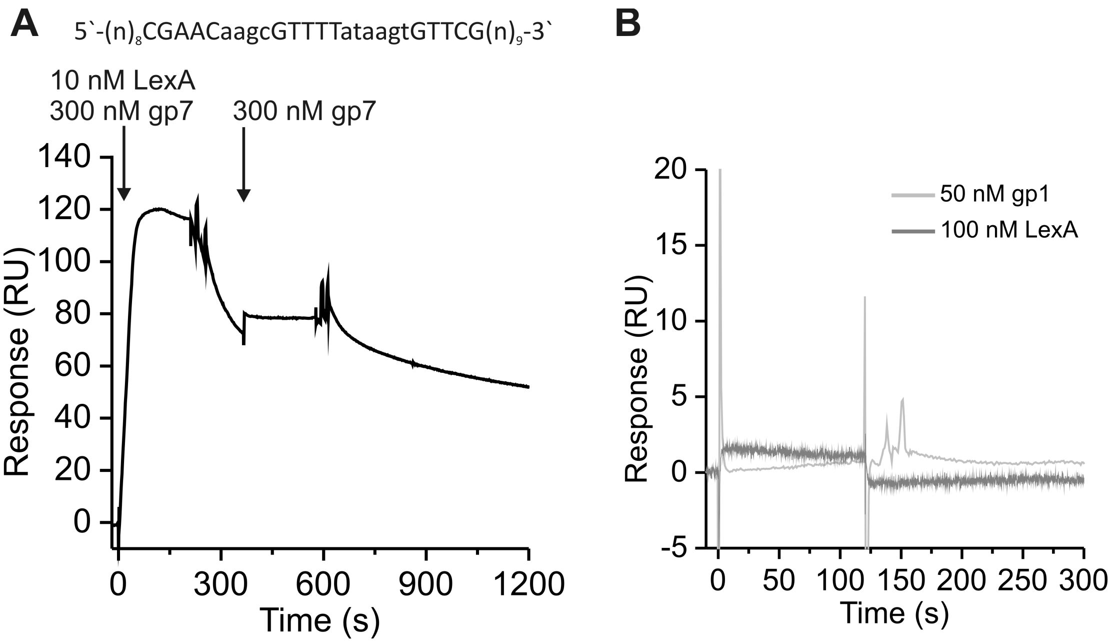

Supplementary Figure S4. SPR analysis of the effect of free gp7 on the LexA-gp7-DNA complex dissociation.

(A) His_6-LexA (10 nM) pre-incubated with His_6-gp7 (300 nM) was injected across a chip-immobilized DNA fragment harboring *dinBox*1 and *dinBox*1b sites (~55 RU) for 210 s at a flow rate of 100 µl/min. Subsequently, in the dissociation phase, His_6-gp7 at a concentration of 300 nM was injected over the nucleoprotein complex for 210 s at 100 µl/min and dissociation was followed for 600 s. The immobilized DNA sequence is presented above the graph. The arrows indicate the time of analyte injection.

(B) His_6-LexA (100 nM) was injected across chip-immobilized His_6-gp7 (96 RU) or His_6-gp1 (50 nM) across chip-immobilized His_6-LexA (2000 RU), both for 120 s at a flow rate of 30 µl/min. The dissociation phase was followed for 180 s.

Experiments in (A) and (B) were performed in duplicate and representative sensorgrams are shown.

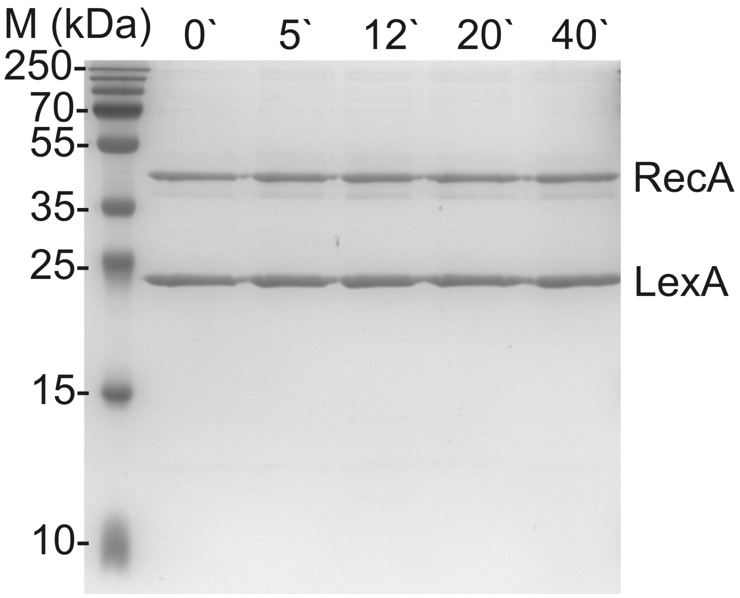

Supplementary Figure S5. Time course of LexA auto-proteolysis in the presence of inactive RecA.

Cleavage of His_6-LexA was monitored at 37°C by SDS-PAGE after the addition of the inactive RecA protein (See also Figure 6C). Intact LexA monomer (LexA) and RecA protein are as marked. The experiment was performed in duplicate and a representative gel is shown.

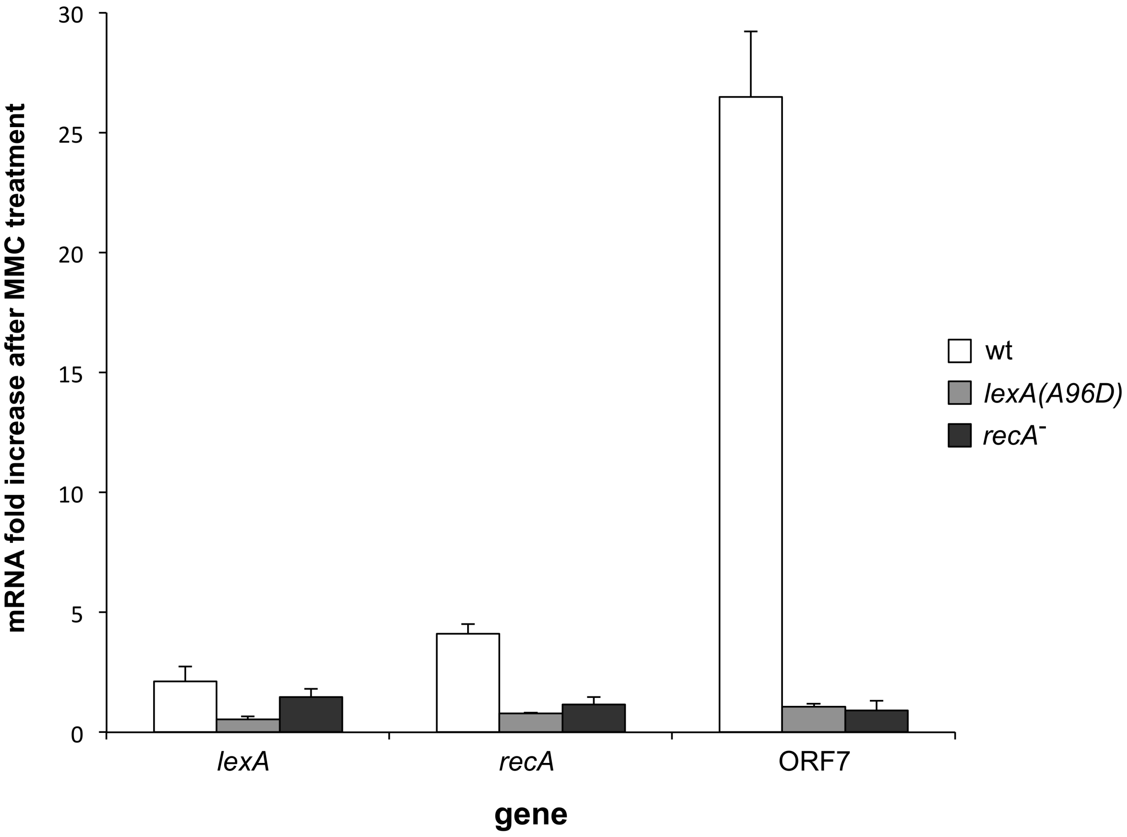

Supplementary Figure S6. ORF7 transcription is highly induced in SOS conditions.

Transcript levels were measured by quantitative RT-PCR in the wild-type, *lexA(A96D)* and *recA*$^-$ lysogens. *lexA* and *recA* were monitored as SOS-inducible reference genes. Fold increase is the amount of mRNA produced in stressed cells divided by the amount of mRNA in the respective untreated cells, relative to the transcript levels from the housekeeping genes *gyrB* and *tufA*. The data shown are the average of three experiments and include the standard deviations.

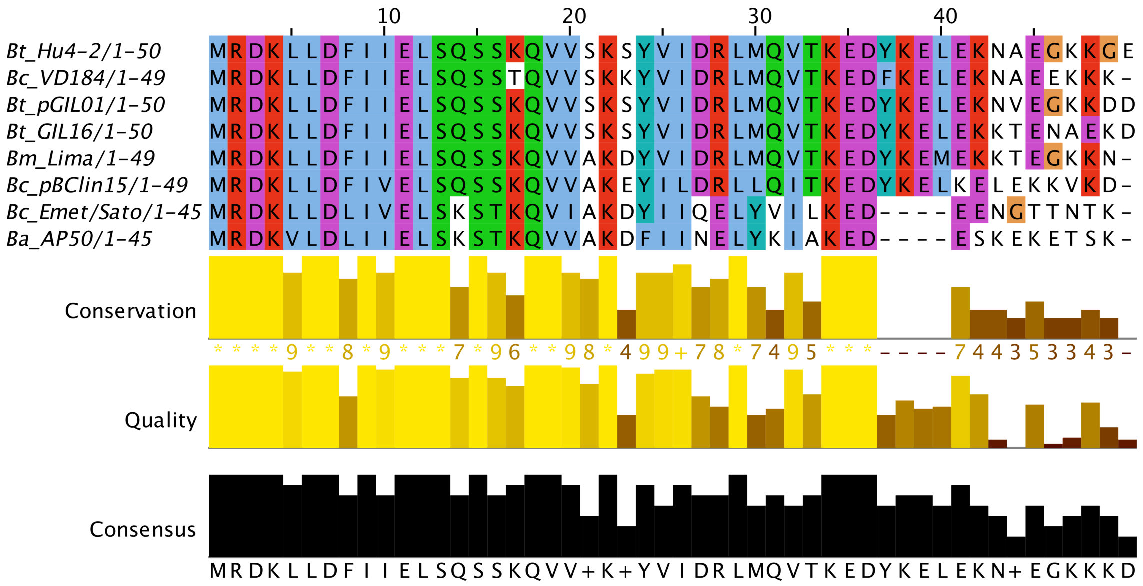

Supplementary Figure S7. Multiple sequence alignment of GIL01-encoded gp7 and tectivirus-derived homologues.

The sequence alignment was generated using ClustalW and visualized with Jalview (1). A tblastn search identified a total of 16 gp7 homologues originating from other tectivirus genomes found in *Bacillus cereus sensu lato* hosts. Homologue sequences were retrieved from Genbank using accession numbers: strain Hu4-2 (AMXT02000219.1), strain VD184 (AHFK01000112.1), bacteriophage pGIL01 (AJ536073.2), bacteriophage GIL16 (NC_006945.1), bacteriophage Lima (KC152964.1), plasmid pBClin15 (AE016878.2), bacteriophage Emet (KC152966.1), bacteriophage Sato (KC152965.1), bacteriophage AP50 (EU408779.1). Gp7 sequences identical to Bt_pGIL01_gp7 were found in plasmid pBTHD789-4 (CP003767.1), strains IBL 4222 (ACNL01000363.1) and ATCC 35646 (AAJM01000106.1), and sequences identical to Bt_GIL16_gp7 were also identified in plasmid pBMBLin15 (CP004882.1), strains ISP2944 (AHEJ01000126.1), HuB13-1 (AHEC01000143.1) and BMG1.7 (AHDQ01000118.1). Bt, *Bacillus thuringiensis*; Bc, *Bacillus cereus*; Bm, *Bacillus mycoides*; Ba, *Bacillus anthracis*.

Supplementary Table S1. Primers and probes used in this study.

Primer name	Primer sequence 5' – 3'	Experiment
136F	GTGTGACAAGATATTCCAA	EMSA and DNase I footprint probes (Figures 2C, 3 and 4)
EMSA3	CCAAGGGTGCTATAATAGGA	
EMSA6	ATATGCACCACCTTCTTTTC	
dinBox1wt_fwd	AAAAAACGAACAAGCGTTTTATAAGTGTTCGGTTTTT	EMSA probes (Figure 2A) and site-directed mutagenesis of *dinBox*es (Figure 1)
dinBox1wt_rev	AAAAACCGAACACTTATAAAACGCTTGTTCGTTTTTT	
dinBox1mut_fwd	AAAAAACGAACAAGC**T**TTTTATAAGTGTTCGGTTTTT	
dinBox1mut_rev	AAAAACCGAACACTTATAAAA**A**GCTTGTTCGTTTTTT	
dinBox1bmut_fwd	AAAAAACGAACAAGCGTTTTATAAGTGTTC**A**GTTTTT	
dinBox1bmut_rev	AAAAAC**T**GAACACTTATAAAACGCTTGTTCGTTTTTT	
dinBox1+1bmut_fwd	AAAAAACGAACAAGC**T**TTTTATAAGTGTTC**A**GTTTTT	
dinBox1+1bmut_rev	AAAAAC**T**GAACACTTATAAAA**A**GCTTGTTCGTTTTTT	
Din1	TGCAAGCTTGACATGTAATAACATTTATG	*P1-P2 lacZ* fusions into pHT304-18Z (Figures 1 and 7)
Din2	GGATCCTCTTTTTATGAACACGCA	
P1mutF	TTCCAAGGGTGCTATAA**C**AGGAAATGAGTT	*P1* -10 sequence site-directed mutagenesis (Figure 1)
P1mutR	AACTCATTTCCT**G**TTATAGCACCCTTGGAA	
P1mut1F	ATATTCCAAGGGTGC**G**ATAACAGGAAATGA	
P1mut1R	CTCATTTCCTGTTAT**C**GCACCCTTGGAATAT	
P1mut2F	AAGATATTCCAAGGGTGCGA**GC**ACAGGAAATGAGTTAAAAAA	
P1mut2R	TTTTTTAACTCATTTCCTGT**GC**TCGCACCCTTGGAATATCTT	
P2mutF	GGTAACTTTCGGGTAATA**C**TGGTAGCAGAG	*P2* -10 sequence site-directed mutagenesis (Figures 1 and 7)
P2mutR	CTCTGCTACCA**G**TATTACCCGAAAGTTACC	
P2mut1F	CTGGTAACTTTCGGG**G**AATACTGGTAGCAGA	
P2mut1R	TCTGCTACCAGTATT**C**CCCGAAAGTTACCAG	
P2mut2F	TTACTGGTAACTTTCGGGGA**GC**ACTGGTAGCAGAGAGGGACA	
P2mut2R	TGTCCCTCTCTGCTACCAGT**GC**TCCCCGAAAGTTACCAGTAA	
DinRecAF	TGCAAGCTTGGATTTTATCATTTATATAAAAAG	*recA-lacZ* fusion into pHT304-18Z (Figure S1)
DinRecAR	TCGGATCCCCATATCTAATGCCGCTT	
DinLexAF	AAGCTTGCATAAATAATTGCATTCAT	*lexA-lacZ* fusion into pHT304-18Z (Figure S1)
DinLexAR	GGATCCGCTTAATAAAGTCGAGAATG	
pQELexF	GGATCCGTGTTAGAAAACATGGAAAAG	Cloning of *lexA* into expression vector pQE-30
pQELexR	AAGCTTTTAATGTAAATCACGATATAC	

Supplementary Table S1. (Continued)

Primer name	Primer sequence 5' – 3'	Experiment
pQEORF1F	GGATCCATGAGTAAACTACTGACTGCG	Cloning of ORF1 into expression vector pQE-30
pQEORF1R	AAGCTTTTAATTGTCCAGTTTATTTT	
pQEORF7F	GGATCCATGCGTGACAAATTGCTCGAC	Cloning of ORF7 into expression vector pQE-30
pQEORF7R	AAGCTTTCAGTCATCCTTCTTCCCC	
RecA_u	CGCGGATCCAGTGATCGTCAAGCGGCATTAG	Cloning of *recA* into expression vector pET8c
RecA_d	CGCACGCGTCTACGTTGAGAAGTTCTAATT	
Wt_u	GTTACTACTCGAGCGTTAAAAAACGAACAAGC GTTTTATAAGTGTTCGGTTTTTGTA	SPR analysis (Figures 2B, 5, 6 and Figures S2, S3, S4)
Wt_d	TACAAAAACCGAACACTTATAAAACGCTTGTTC GTTTTTTAA	
B11_u	GTTACTACTCGAGCGTTAAAAAACGAACAAGCT TTTTATAAGTGTTCGGTTTTTGTA	
B11_d	TACAAAAACCGAACACTTATAAAAAGCTTGTTC GTTTTTTAA	
B21_u	GTTACTACTCGAGCGTTAAAAAACGAACAAGC GTTTTATAAGTGTTCAGTTTTTGTA	
B21_d	TACAAAAACTGAACACTTATAAAACGCTTGTTC GTTTTTTAA	
B12_u	GTTACTACTCGAGCGTTAAAAAACTAACAAGCT TTTTATAAGTGTTCGGTTTTTGTA	
B12_d	TACAAAAACCGAACACTTATAAAAAGCTTGTTA GTTTTTTAA	
B22_u	GTTACTACTCGAGCGTTAAAAAACGAACAAGC GTTTTATAAGTATTCAGTTTTTGTA	
B22_d	TACAAAAACTGAATACTTATAAAACGCTTGTTC GTTTTTTAA	
Ns_u	GTTACTACTCGAGCGTTAAAAAACTAACAAGCT TTTTATAAGTATTCAGTTTTTGTA	
Ns_d	TACAAAAACTGAATACTTATAAAAAGCTTGTTA GTTTTTTAA	
lexA_u	GTTACTACTCGAGCGGCCATTTGTTCGAACTTA TGTTTGTAGAATGTTTTTTCAC	
lexA_d	GTGAAAAAACATTCTACAAACATAAGTTCGAAC AAATGGC	
lexAns_u	GTTACTACTCGAGCGGCCATTTGTTCTAGCTTA TGTGTGTAGAATGTTTTTTCAC	
lexAns_d	GTGAAAAAACATTCTACACACATAAGCTAGAAC AAATGGC	
S1	CGCTCGAGTAGTAAC-Bio	
qPCR_orf7 F	GCGTGACAAATTGCTCGACT	Quantitative RT-PCR experiments (Figure S6)
qPCR_orf7 R	AGTCATCCTTCTTCCCCTCCA	
qPCR_lexA F	CGAAACGCCAGCAAGACATT	
qPCR_lexA R	ATGGCACGTGGTTTTGTTGG	
qPCR_recA F	ACGTGTGAGTCACCCATGTC	
qPCR_recA R	TCACAGCCTGATACAGGGGA	
qPCR_gyrB F	TGAAGCGAGGACGATTACGG	
qPCR_gyrB R	AAGCACTCTTTCGGCGAGTT	
qPCR_tufA F	AAATCGACGCTGCTCCAGAA	
qPCR_tufA R	CATAGTCAGCGTGACCTGGG	

Restriction sites are underlined, the primer extension annealing to the SPR chip-immobilized S1 primer is double underlined and important bases for LexA binding are underlined with dashed lines. Substituted nucleotides are shown in bold and ‘Bio’ denotes the biotin label.
